# Continuous-Flow Synthesis of Thioureas, Enabled by Aqueous Polysulfide Solution

**DOI:** 10.3390/molecules26020303

**Published:** 2021-01-08

**Authors:** András Gy. Németh, Renáta Szabó, György Orsy, István M. Mándity, György M. Keserű, Péter Ábrányi-Balogh

**Affiliations:** 1 Medicinal Chemistry Research Group, Research Centre for Natural Sciences, H-1117 Budapest, Hungary; nemeth.andras.gyorgy@ttk.hu (A.G.N.); szaboreni287@gmail.com (R.S.); 2 Lendület Artificial Transporters Research Group, Research Centre for Natural Sciences, H-1117 Budapest, Hungary; orsy.gyorgy@ttk.hu (G.O.); mandity.istvan@ttk.mta.hu (I.M.M.)

**Keywords:** continuous flow, sulfur, aqueous polysulfide solution, thiourea, multicomponent reaction

## Abstract

We have developed the continuous-flow synthesis of thioureas in a multicomponent reaction starting from isocyanides, amidines, or amines and sulfur. The aqueous polysulfide solution enabled the application of sulfur under homogeneous and mild conditions. The crystallized products were isolated by simple filtration after the removal of the co-solvent, and the sulfur retained in the mother liquid. Presenting a wide range of thioureas synthesized by this procedure confirms the utility of the convenient continuous-flow application of sulfur.

## 1. Introduction

In the last two decades, continuous-flow (CF) synthesis has become a powerful and versatile tool in synthetic organic chemistry. The enhanced mixing properties, heat, and mass transfer of CF systems lead to more precise regulation of the reaction conditions, and thus, better reproducibility and selectivity can be realized than in batch processes [1,2]. Notably, CF chemistry has been used in hazardous reactions (nitration, halogenation, reaction with organolithium reagents and azides, etc.) [3,4] and in synthetic methods requiring a high temperature and pressure [5,6,7], as well. In addition, it enables cleaner reaction profiles with better product–side-product ratios, and reaction pathways that could hardly be realized in batch processes (e.g., use of highly reactive peptidyl donors) [8,9,10]. Moreover, CF techniques are suitable for multistep [11,12,13,14] and automated synthetic processes, which is a rapidly growing field in modern organic chemistry [15,16,17].

**Scheme 1:**
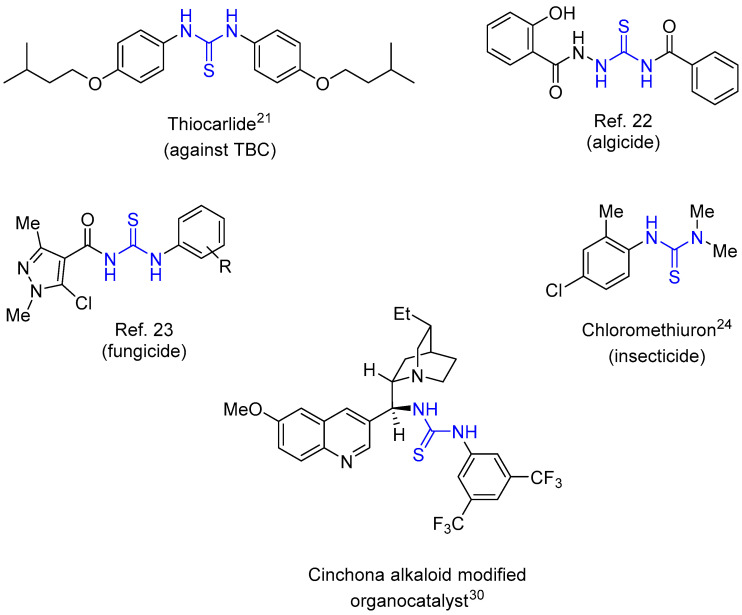
Examples of important thioureas.

Sulfur-containing compounds are widely known as biologically active molecules [18] and functional organic materials [19,20]. Thioureas, in particular, are used as pharmaceutical and agrochemical intermediates or active ingredients represented by the marketed drug thiocarlide [21], and by algicides [22], fungicides [23], and the insecticide chloromethiuron [24]. In addition, they are key intermediates of nitrogen- and sulfur-containing compounds, especially pharmacologically relevant heterocycles [25,26,27,28,29]. Notably, in the last two decades, thioureas were also applied as highly selective and efficient organocatalysts [30,31,32,33,34] (Scheme 1). Given the wide utility of thioureas, their clean and efficient synthesis is of high interest.

Elemental sulfur is a bench-stable, environmentally benign, inexpensive, and nontoxic reagent for sulfuration, and offers an atom-economical and safe alternative to incorporate the sulfur atom into products [35]. In the last two decades, the uses of elemental sulfur in chemical reactions have emerged greatly, leading to, in particular, many innovative, multicomponent, and one-pot procedures [36,37,38,39,40,41,42]. Notably, a handful of thiourea syntheses have been developed as well [34,43,44,45]. Most synthetic methods require chromatographic purification and apply sulfur in solid form, which makes the transfer of these reactions into CF processes inconvenient. Nonetheless, Shavel et al. realized the continuous production of Cu_2_ZnSnS_4_ nanoparticles at 300 °C, starting from metal complexes and sulfur [46]. Organic reactions, however, require milder conditions to provide selectivity and maintain the stability of the compounds. Recently, we prepared aqueous polysulfide solutions from elemental sulfur with organic and inorganic bases and used it efficiently for the mild multicomponent preparation of thioureas starting from isocyanides and amidines or amines [47]. Herein, as a model study, we report the CF synthesis of thioureas using elemental sulfur under homogeneous conditions.

## 2. Results

**Figure 1:**
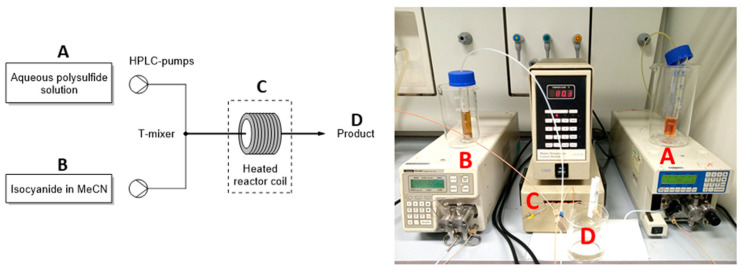
Image of the experimental setup.

First, we performed the model reaction of 2,6-dimethylphenyl isocyanide (**1**) and the polysulfide solution made of 1,8-diazabicyclo[5.4.0]undec-7-ene (DBU, **3**) and elemental sulfur. In this reaction, the base (**3**) opened the S_8_-crown, resulting in polysulfide anions. These become the reactive agents attacking the isocyanide, leading to isothiocyanates in situ, which was able to acylate a nucleophilic amine in its close surroundings. In this model reaction, the open form of DBU acted as the corresponding nucleophile [47,48,49,50,51]. We applied two HPLC pumps to provide the feed for the solution of the isocyanide in acetonitrile (0.2 M), and the aqueous polysulfide solution containing DBU (1.0 M for the base, 0.4 M for the sulfur). The two inputs met in a T-mixer right before the heated reactor oven, and the output was collected in a flask (Figure 1).

Applying a residence time of 26 s at 60 °C enabled a conversion of 36% for the isocyanide, monitored by HPLC–MS at 190 nm (Table 1, Entry 1). This was improved to 50% by a longer residence time of 66 s (Entry 2), and to 62% by maintaining the reaction temperature at 80 °C (Entry 3). Eventually, increasing the residence time gradually to 6.5 min enabled the practically full conversion of **1** (Entries 4–7). At 100 °C, we observed the appearance of side products, and thus, kept the reaction temperature at 80 °C. We removed the acetonitrile in vacuo, and the product crystallized from water. We were able to isolate the pure thiourea **4** in 88% yield by simple filtration, while the excess of DBU and polysulfide anions were washed away by water (Table 1).

With the optimized reaction conditions in hand, we planned to investigate the scope and limitations of the reaction. First, we applied different amidine type bases (**9** and **10**) and isocyanides of a broad structural diversity (**1** and **5–8**) using the same experimental setup (Figure 1, Table 2). Using **1** together with the polysulfide solutions made of commercially available amidines 1,5-diazabicyclo[4.3.0]non-5-ene (DBN, **9**) and 1,5,7-triazabicyclo[4.4.0]dec-5-ene (TBD, **10**), we isolated the thiourea **11** in 90% yield and the tetrahydropyrimidin-2(1*H*)-one derivative **12** in 56% yield (Entries 1 and 2). The pyridine and the quinoline-containing thioureas **13** and **14** were isolated in 67% and 68% yields, respectively (Entries 3 and 4). The aliphatic phenethyl isocyanide **7** and 2-(indole-3-yl)ethyl isocyanide **8** provided the corresponding products (**15** and **16**) in slightly lower 40% and 54% yields, respectively (Entries 5 and 6). Notably, after the removal of the acetonitrile in vacuo, all thioureas crystallized from water and were isolated by simple filtration.

Considering bases resistant to acylating agents, the reaction may provide virtually any desired thiourea. Recently, we used *N*,*N*,*N′*,*N″*,*N″*-pentamethyldiethylenetriamine (PMDTA)-based aqueous polysulfide solution for the synthesis of versatile thiourea derivatives [47]. Following the extension of the reaction, we applied this aqueous polysulfide solution in the reaction with **1** and benzylamine **19** in the continuous stream. This resulted in the formation of thiourea **26** in excellent, 96% yield, with an extended 42 min of residence time, which was necessary for the complete conversion of the isocyanide (Table 3, Entry 1). In this setup, the solution of the isocyanide and the amine in acetonitrile provided one feed, and the other feed contained the aqueous polysulfide solution. The 3-isocyano quinoline **6** reacted well, leading to the formation of the thiourea **27** in 92% yield (Entry 2). Next, we applied aliphatic isocyanides **7**, **17**, and **18**, which provided the thioureas **28**, **29**, and **30** in moderate yields (49%, 42%, and 42%, respectively, Entries 3–5). These results clearly indicate the convenience of aromatic isocyanides over aliphatic ones. The phenylethylamine and morpholine derivatives **31** and **32** were isolated in 96% and 76% yields, respectively (Entries 6 and 7). Notably, due to the precipitation of the product from the water–acetonitrile mixture, in the case of aniline **22**, we applied 2-methyltetrahydrofuran as a co-solvent and isolated the biaryl thiourea **33** in 70% yield (Entry 8). After the removal of the polysulfide solution by filtration, the aniline was washed away with 1.0 M aq. HCl. The 4-methyl and the halogen-substituted biaryl thioureas **34**–**36** were isolated in 79%, 39%, and 36% yields, respectively (entries 9–11), showing favor of the reaction to the electron donor substrates.

## 3. Materials and Methods

### 3.1. General

All melting points were determined on a Jasco SRS OptiMelt apparatus and are uncorrected. ^1^H-NMR and ^13^C-NMR spectra were recorded in DMSO-*d_6_* or CDCl_3_ solution at room temperature, on a Varian Unity Inova 500 spectrometer (Bruker Corp., Oxford, UK) (500 and 125 MHz for ^1^H-NMR and APT-NMR spectra, respectively), with the residual solvent signal as the lock and TMS (tetramethylsilane) as the internal standard. Chemical shifts (δ) and coupling constants (J) are given in ppm and Hz, respectively. HPLC–MS measurements were performed using a Shimadzu LCMS-2020 (Shimadzu Corp., Kyoto, Japan) device, equipped with a Reprospher (Altmann Analytik Corp., München, Germany) 100 C18 (5 µm; 100 × 3 mm) column and a positive/negative double ion source (DUIS±) with a quadrupole MS analyzer in a range of 50–1000 *m/z*. The samples were eluted with gradient elution, using eluent A (0.1% formic acid in water) and eluent B (0.1% formic acid in acetonitrile). The flow rate was set to 1.5 mL/min. The initial condition was 5% eluent B, followed by a linear gradient to 100% eluent B by 1.5 min; from 1.5 to 4.0 min, 100% eluent B was retained; and from 4 to 4.5 min, it went back by a linear gradient to 5% eluent B, which was retained from 4.5 to 5 min. The column temperature was kept at room temperature, and the injection volume was 1–10 µL. The purity of the compounds was assessed by HPLC with UV detection at 215 and 254 nm; all starting compounds were known, purchased, or synthetically feasible, and >95% pure. In the CF system, the stream of the solution of the starting materials was provided by HPLC pumps (JASCO model PU–2080), and the tubing (BGB, 1/16″ OD × 0.50 mm or 1.00 mm ID, 10 m) was placed in a Carlo Erba HRGC 5300 oven. Compounds not precedented in the literature were characterized by ^1^H-NMR and ^13^C-NMR, HRMS and if were obtained in solid form by melting point. For known compounds ^1^H-NMR spectra and melting points were measured. All spectra and data is available in the Supplementary Materials.

### 3.2. General Procedure for the Preparation of the Aqueous Solution of Polysulfide Anions

Sulfur (32 mg, 1.0 mmol) was added to a mixture of 1,8-diazabicyclo[5.4.0]undec-7-ene (373 µL, 2.5 mmol) and water (2.13 mL), and stirred vigorously at 60 °C until the complete dissolution of the sulfur (Table 4).

### 3.3. General Procedure for the CF Synthesis of Thioureas ***11***–***16***

Isocyanide (**1**, **5**–**8**; 3.0 mmol) was dissolved in MeCN, then filtered through a 0.45 µm pore-sized syringe filter to provide Feed A (0.2 M in MeCN). The aqueous solution of sulfur and the appropriate amidine (**3**, **9**, **10**) was used for Feed B (1.0 M base, 0.4 M sulfur). Feeds A and B were pumped into a T-mixer at room temperature at flow rates of 0.15 mL min^−1^ each. The mixture passed through a reaction coil at 80 °C in 6.5 min, then collected in an Erlenmeyer flask. Altogether, 0.5 mmol of product was collected (calculated on the used isocyanide), the acetonitrile was evaporated in vacuo, and the product was filtered and washed with water to provide thioureas **11**–**16**.

### 3.4. General Procedure for the CF Synthesis of Thioureas ***26***–***36***

Isocyanide (**1**, **6**, **7**, **17**, **18**; 3.0 mmol) and amine (**19**–**25**, 4.5 or 9.0 mmol) was dissolved in MeCN, then filtered through a 0.45 µm pore-sized syringe filter to provide Feed A (0.2 M isocyanide and 0.3 or 0.6 M amine in MeCN). The aqueous polysulfide solution made of PMDTA and elemental sulfur was used for Feed B (1.0 M PMDTA, 0.4 M sulfur). Feeds A and B were pumped into a T-mixer at room temperature at flow rates of 0.10 mL min^−1^ each. The mixture passed through a reaction coil at 80 °C in 42 min, then collected in an Erlenmeyer flask. Altogether, 0.5 mmol of product was collected (calculated on the used isocyanide), the acetonitrile was evaporated in vacuo, and the product was filtered and washed with water to provide thioureas **26**–**36**.

## 4. Conclusions

Starting from our former batch procedure, we developed a new continuous-flow synthesis of thioureas by the multicomponent reaction of aqueous polysulfide solution, isocyanides, and amidines or amines. We have shown the convenient continuous-flow application of elemental sulfur and explored the scope and limitations of the procedure. Notably, the products were isolated by simple filtration, and no further purification was necessary. We believe that this approach widens the synthetic toolbox for the development of new methods using polysulfide solution for the incorporation of sulfur into organic molecules.

## Figures and Tables

**Table 1:** Optimization of the reaction conditions for the synthesis of thioureas **4** under CF conditions.

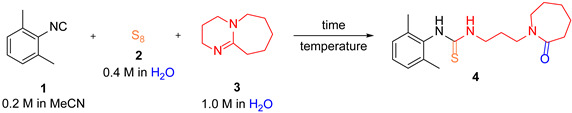				
Entry ^a^	T [°C]	Flow Rate [mL min^−1^]	Residence Time	HPLC Conversion 4/1 ^b,c^ [%]
1	60	1.0	26 s	36/64
2	60	0.4	1 min 6 s	50/50
3	80	0.4	1 min 6 s	62/38
4	80	0.2	2 min 12 s	84/16
5	80	0.6	3 min 16 s	93/7
6	80	0.4	4 min 54 s	98/2
7	80	0.3	6 min 32 s	99/1 (88)

^a^ Reaction conditions: isocyanide **1** (0.2 M in acetonitrile), polysulfide solution (1.0 M **3**, 0.4 M sulfur in water); ^b^ Conversion is based on UV–VIS absorbance at 190 nm; ^c^ Isolated yield in parentheses for 0.5 mmol scale.

**Table 2:** Scope of isocyanides and amidines.

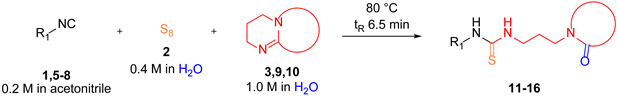				
Entry ^a^	Isocyanide	Amine	Product	Yield [%] ^b^
1	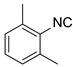 **1**	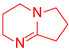 **9**	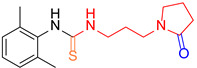 **11**	90
2		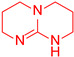 **10**	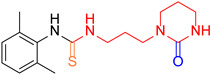 **12**	56
3	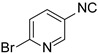 **5**	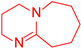 **3**	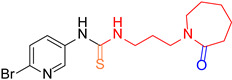 **13**	67
4	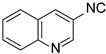 **6**		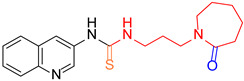 **14**	68
5	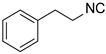 **7**		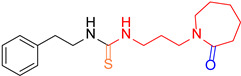 **15**	40
6	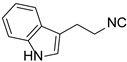 **8**		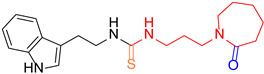 **16**	54

^a^ Reaction conditions: isocyanide **1a**–**e** (0.2 M in acetonitrile), polysulfide solution (1.0 M **3b**,**c**, 0.4 M sulfur in water); ^b^ 0.5 mmol scale.

**Table 3:** Scope of isocyanides and amines for the synthesis of thioureas **26**–**36**.

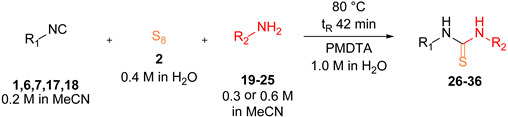				
Entry ^a^	Isocyanide	Amine	Product	Yield [%] ^d^
1	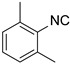 **1**	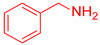 **19**	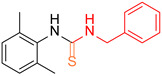 **26**	96
2	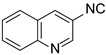 **6**		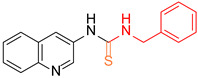 **27**	92
3	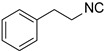 **7**		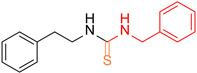 **28**	49
4	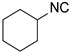 **17**		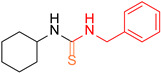 **29**	42
5	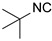 **18**		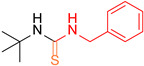 **30**	42
6	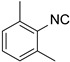 **1**	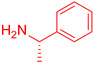 **20**	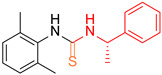 **31**	96
7 ^b^	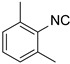 **1**	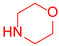 **21**	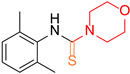 **32 ^b^**	76
8 ^b,c^		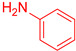 **22**	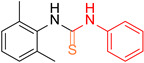 **33 ^b,c^**	70
9 ^b^		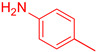 **23**	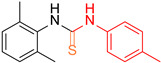 **34 ^b^**	79
10 ^b^		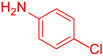 **24**	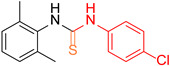 **35 ^b^**	39
11 ^b^		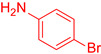 **25**	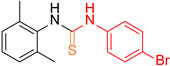 **36 ^b^**	36

^a^ Reaction conditions: isocyanide **1**,**6**,**7**,**17**,**18** and amine **19**–**25** (0.2 M isocyanide and 0.3 M amine in acetonitrile), polysulfide solution (1.0 M PMDTA and 0.4 M sulfur in water); ^b^ 0.6 M for the amine in acetonitrile; ^c^ 2-Methyltetrahydrofuran was used as a co-solvent instead of MeCN; ^d^ 0.5 mmol scale.

**Table 4:** Preparation of polysulfide solutions, according to the general procedure.

Amine	Sulfur [mg, mmol]	Amine [µL or mg, mmol]	Water [mL]	T [°C]
1,8-diazabicyclo[5.4.0]undec-7-ene	32, 1.00	373 µL, 2.50	2.13	60
1,5-Diazabicyclo[4.3.0]non-5-ene		310 µL, 2.50	2.19	60
1,5,7-Triazabicyclo[4.4.0]dec-5-ene		348 mg, 2.50	2.50	60
7-Methyl-1,5,7-triazabicyclo[4.4.0]dec-5-ene		360 µL, 2.50	2.17	60
*N*,*N*,*N′*,*N″*,*N″*-Pentamethyldiethylenetriamine		522 µL, 2.50	1.98	70

## Data Availability

All data supporting this study is available in the manuscript and the Supplementary Materials.

## Supplementary Materials

The following are available online: general procedures for the isocyanides, characterization data, and NMR spectra.

## Sample Availability

Samples of the compounds are available upon request from the corresponding authors.
