# Supplementary material for: Continuous-Flow Synthesis of Thioureas, Enabled by Aqueous Polysulfide Solution
Source: Molecules. 2021 Jan 8;26(2):303. doi: 10.3390/molecules26020303 (PMC7827778; doi:10.3390/molecules26020303)
Supplement: Supplementary file 1 [file molecules-26-00303-s001.pdf]

Supporting information

Continuous-Flow Synthesis of Thioureas Enabled by Aqueous Polysulfide Solution

András Gy Németh [1], Renáta Szabó [1], György Orsy [2], István M. Mándity [2], György M. Keserű [1,*] and Péter Ábrányi-Balogh [1,*]

[1] Medicinal Chemistry Research Group, Research Centre for Natural Sciences, Magyar tudósok krt. 2, H-1117 Budapest, Hungary; nemeth.andras.gyorgy@ttk.hu (A.G.N); szaboreni287@gmail.com (R.S.)
[2] Artificial Transporters Research Group, Research Centre for Natural Sciences, Magyar tudósok krt. 2, H-1117 Budapest, Hungary; orsy.gyorgy@ttk.hu (G.O.); mandity.istvan@ttk.mta.hu (I.M.M.)
* Correspondence: keseru.gyorgy@ttk.hu (G.M.K.); abranyi-balogh.peter@ttk.hu (P.A.-B.)

Table of Contents

Batch results for the synthesis of thioureas S2

Experimental data S3

^{1}H and ^{13}C NMR spectra S7

References S34

Table S1. Batch results for compounds **4**, **11–14**, **16**, **26** and **29–36** from reference 1.

Product[a]	Yield [%][b]	Product[a]	Yield [%][b]
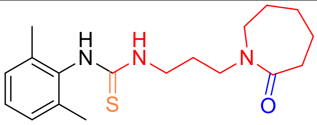 **4** (0.5 h)	91[c]	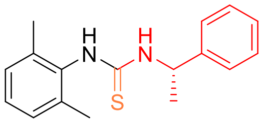 **31** (0.5 h)	84[e]
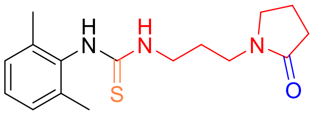 **11** (1 h)	92[c]	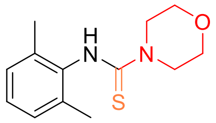 **32** (0.5 h)	74[e]
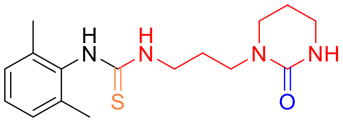 **12** (2 h)	62[c]	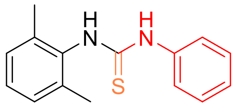 **33** (0.5 h)	74[e]
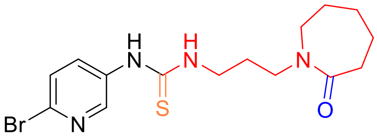 **13** (0.5 h)	67[c]	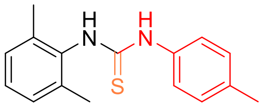 **34** (1 h)	81[e]
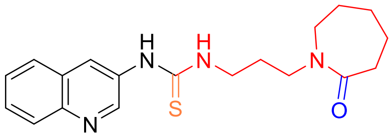 **14** (0.5 h)	72[c]	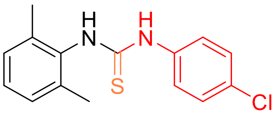 **35** (1 h)	76[e]
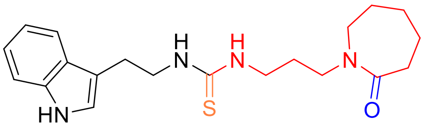 **16** (1 h)	50[c]	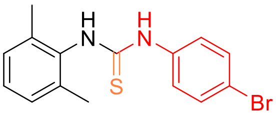 **36** (1 h)	77[e]
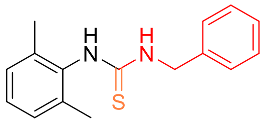 **26** (0.5 h)	89[d]		
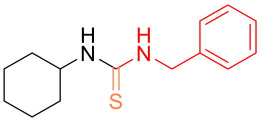 **29** (2 h)	81[d]		
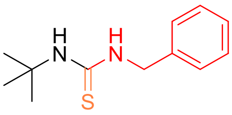 **30** (2 h)	89[d]		

[a]Reaction time in parentheses. [b]Isolated yields. [c]Reaction conditions: isocyanide (0.5 mmol), polysulfide solution (1.0 M amidine/0.4 M S_8, 2.5 mL), 60 °C, water. [d]Reaction conditions: isocyanide (0.50 mmol), amine (0.75 mmol), polysulfide solution (1.0 M PMDTA/0.4 M S_8, 2.5 mL), 60 °C, water. [e]Reaction conditions: same as the previous conditions at 80 °C.

All of the compounds except **15**, **27** and **28** are known in the literature, and if not indicated otherwise, have been characterized in reference 1.

General procedure for the synthesis of formamides:

To formic acid (1.66 mL, 44 mmol) at 0 °C acetic anhydride (3.40 mL, 36 mmol) was added, then the mixture was heated up to 60 °C for 2 h. After cooling to 0 °C the reaction mixture was diluted with tetrahydrofuran (15 mL) and the solution of the amine (10 mmol) in tetrahydrofuran (15 mL) was added carefully. After the complete consumption of the amine followed by TLC, the volatiles were evaporated and flash column chromatography was applied if necessary providing the formamides 89%-quantitative yield.

***N*-(6-bromopyridin-3-yl)formamide (S1)**: Pale yellow crystals (2.01 g, quant.), m.p. 158–159 °C (acetic acid–formic acid); ^{1}H-NMR: (500 MHz, DMSO-d_6, mixture of E/Z isomers) δ 10.41 ((two signals: 10.52 (bs), 10.29 (d, *J* = 10.2 Hz)), 1H), 8.71 ((two signals: 8.81 (d, *J* = 10.8 Hz), 8.60 (d, *J* = 2.3 Hz)), 1H), 8.33 ((two signals: 8.37 (s), 8.29 (s)), 1H), 7.79 ((two signals: 7.97 (dd, *J* = 8.6 Hz, *J* = 2.5 Hz), 7.60 (m)), 1H), 7.60–7.58 (m, 1H) ppm.

***N*-(quinolin-3-yl)formamide (S2)**: Compound have been characterized in ref.[2] The observed ^{1}H NMR spectrum was consistent with the previously reported data (although the solvent was MeOD); White solid (1.54 g, 89%), m.p. 160–161 °C (acetic acid–formic acid, ref.[2] chloroform–methanol 159.5–160.5 °C); ^{1}H-NMR: (500 MHz, DMSO-d_6, mixture of E/Z isomers, ref.[2]) δ 10.57 ((two signals: 10.66 (bs), 10.48 (bs)), 1H), 8.94 ((two signals: 8.96 (bs), 8.92 (d, *J* = 2.5 Hz)), 1H), 8.76 ((two signals: 8.82 (d, *J* = 2.4 Hz), 8.70 (d, *J* = 2.3 Hz)), 1H), 8.31 ((two signals: 8.46 (s), 8.15 (d, *J* = 2.1 Hz)), 1H), 7.97–7.87 (m, 2H), 7.67–7.56 (m, 2H) ppm.

***N*-(2-(1H-indol-3-yl)ethyl)formamide (S3)**: Compound have been characterized in ref.[3] The observed ^{1}H NMR spectrum was consistent with the previously reported data; Yellow oil (1.86 g, 99%); ^{1}H-NMR: (500 MHz, $CDCl_3$, mixture of E/Z isomers, ref. [3]) δ 8.35 (bs, 1H), 7.95 ((two signals: 8.06 (bs), 7.84 (d, *J* = 12.0 Hz)), 1H), 7.57 ((two signals: 7.59 (d, *J* = 7.9 Hz), 7.55 (d, *J* = 7.9 Hz)), 1H), 7.36 (d, *J* = 8.2 Hz, 1H), 7.21 (t, *J* = 8.0 Hz, 1H), 7.14–7.11 (m, 1H), 6.99 ((two signals: 7.01 (bs), 6.97 (bs)), 1H), 5.85 ((two signals: 5.95 (bs), 5.75 (bs)), 1H), 3.56 ((two signals: 3.63 (q, *J* = 6.5 Hz), 3.48 (q, *J* = 6.4 Hz)), 2H), 3.00–2.93 (m, 2H) ppm.

General procedure for the synthesis of isocyanides 5, 6 and 8:

To a solution of the formamide (5 mmol) in tetrahydrofuran (10 mL) and triethylamine (5.58 mL, 40 mmol) was added phosphoryl chloride (0.93 mL, 10 mmol) dropwise at 0 °C. The resulted mixture was stirred at either room temperature (isocyanide **5** and **8**) or reflux temperature (isocyanide **6**) until the consumption of the formamide followed by TLC. Afterwards, aqueous sodium hydrogencarbonate (15 mL) was added slowly under ice cooling, then the reaction mixture was extracted with dichloromethane (2 × 15 mL) and the organic layer was washed with brine (15 mL). Evaporation of the solvent followed by purification by flash column chromatography in hexane-ethyl acetate on aluminium oxide provided the isocyanide **5, 6** and **8** in 54–59% yield.

2-Bromo-5-isocyanopyridine (5): White crystals (490 mg, 54%), m.p. 113–114 °C (decomp., hexane–ethyl acetate); ^{1}H-NMR: (500 MHz, $CDCl_3$) δ 8.45 (d, *J* = 1.3 Hz, 1H), 7.58–7.53 (m, 2H) ppm.

3-Isocyanoquinoline (6): Compound have been characterized in ref.[4] The observed ^{1}H NMR spectrum was consistent with the previously reported data; Pale yellow crystals (455 mg, 59%), m.p. 83–84 °C (hexane–dichloromethane); ^{1}H-NMR: (500 MHz, DMSO-d_6, ref.[4]) δ 8.90 (d, *J* = 2.3 Hz, 1H), 8.19 (d, *J* = 1.9 Hz, 1H), 8.15 (d, *J* = 8.5 Hz, 1H), 7.86–7.80 (m, 2H), 7.66 (td, J_1 = 7.6 Hz, J_2 = 1.0 Hz, 1H) ppm.

3-(2-Isocyanoethyl)-1H-indole (8): Pale yellow crystals (502 mg, 59%), m.p. 70–71 °C (hexane–ethyl acetate); ^{1}H-NMR: (500 MHz, $CDCl_3$) δ 8.17 (bs, 1H), 7.59 (d, *J* = 7.8 Hz, 1H), 7.39 (d, *J* = 8.2 Hz, 1H), 7.26 (td, J_1 = 7.6 Hz, J_2 = 0.9 Hz, 1H), 7.19 (td, J_1 = 7.5 Hz, J_2 = 0.9 Hz, 1H), 7.11 (d, *J* = 2.3 Hz, 1H), 3.67 (t, *J* = 7.0 Hz, 1H), 3.17 (t, *J* = 6.9 Hz, 1H) ppm.

(2-Isocyanoethyl)benzene (7): Compound have been characterized in ref.[5] The observed ^{1}H NMR spectrum was consistent with the previously reported data (only the coupling constants are different); To formic acid (1.66 mL, 44 mmol) at 0 °C acetic anhydride (3.40 mL, 36 mmol) was added, then the mixture was heated up to 60 °C for 2 h. After cooling to 0 °C the reaction mixture was diluted with tetrahydrofuran (15 mL) and the solution of the amine (10 mmol) in tetrahydrofuran (15 mL) was added carefully. After the complete consumption of the amine followed by TLC, the volatiles were evaporated. The crude product was dissolved in tetrahydrofuran (20 mL) and triethylamine (11.2 mL, 80 mmol), then phosphoryl chloride (1.86 mL, 20 mmol) was added dropwise at 0 °C. The resulted mixture was stirred at room temperature until the consumption of the formamide followed by TLC. Afterwards, aqueous sodium hydrogencarbonate (30 mL) was added slowly under ice cooling, then the reaction mixture was extracted with dichloromethane (2×30 mL) and the organic layer was washed with brine (30 mL). Evaporation of the solvent followed by purification by flash column chromatography in hexane-ethyl acetate on aluminium oxide provided the isocyanide 7 in 65% yield (850 mg). Yellow oil; ^{1}H-NMR: (500 MHz, $CDCl_3$, ref. [5]) δ 7.36–7.23 (m, 5H), 3.61 (t, *J* = 7.1 Hz, 2H), 2.99 (t, *J* = 7.1 Hz, 2H) ppm.

1-(2,6-Dimethylphenyl)-3-(3-(2-oxoazepan-1-yl)propyl)thiourea (4): White solid, m.p. 163–164 °C (water); ^{1}H-NMR: (500 MHz, $CDCl_3$) δ 7.25 (bs, 1H), 7.21–7.14 (m, 3H), 6.38 (bs, 1H), 3.57 (t, 2H, *J* = 6.0 Hz), 3.29–3.27 (m, 4H), 2.41–2.38 (m, 2H), 2.27 (s, 6H), 1.77–1.60 (m, 8H) ppm.

1-(2,6-Dimethylphenyl)-3-(3-(2-oxopyrrolidin-1-yl)propyl)thiourea (11): White solid, m.p. 154–155 °C (water); ^{1}H-NMR: (500 MHz, $CDCl_3$) δ 7.27 (bs, 1H), 7.22–7.14 (m, 3H), 6.32 (bs, 1H), 3.54 (bs, 2H), 3.35 (t, 2H, *J* = 7.0 Hz), 3.18 (bs, 2H), 2.32–2.24 (m, 2H), 2.27 (s, 6H), 2.01–1.95 (m, 2H), 1.79–1.76 (m, 2H) ppm.

1-(2,6-Dimethylphenyl)-3-(3-(2-oxotetrahydropyrimidin-1(*2H*)-yl)propyl)thiourea (12): White solid, m.p. 142–143 °C (water); ^{1}H-NMR: (500 MHz, $CDCl_3$) δ 7.27 (bs, 1H), 7.18–7.11 (m, 3H), 6.67 (bs, 1H), 4.50 (bs, 1H), 3.59–3.56 (m, 2H), 3.20–3.18 (m, 6H), 2.25 (s, 6H), 1.88–1.86 (m, 2H), 1.73 (p, 2H, *J* = 6.1 Hz) ppm.

1-(6-Bromopyridin-3-yl)-3-(3-(2-oxoazepan-1-yl)propyl)thiourea (13): White solid, m.p. 182–183 °C (water); ^{1}H-NMR: (500 MHz, DMSO-d_6) δ 9.77 (bs, 1H), 8.40 (d, *J* = 2.7 Hz, 1H), 8.01 (bs, 1H), 7.94 (d, *J* = 6.9 Hz, 1H), 7.56 (d, *J* = 8.6 Hz, 1H), 3.36–3.30 (m, 6H), 2.42 (t, *J* = 5.6 Hz, 2H), 1.72–1.54 (m, 8H) ppm.

1-(3-(2-Oxoazepan-1-yl)propyl)-3-(quinolin-3-yl)thiourea (14): Beige crystals, m.p. 134–135 °C (water); ^{1}H-NMR: (500 MHz, $CDCl_3$) δ 9.18 (bs, 1H), 8.82 (s, 1H), 8.37 (s, 1H), 7.96 (d, *J* = 8.4 Hz, 1H), 7.85 (bs, 1H), 7.71 (d, *J* = 8.1 Hz, 1H), 7.55 (t, *J* = 7.9 Hz, 1H), 7.43 (d, *J* = 7.7 Hz, 1H), 3.62 (d, *J* = 5.4 Hz, 2H), 3.36 (t, *J* = 6.1 Hz, 2H), 3.26 (t, *J* = 4.5 Hz, 2H), 2.39 (t, *J* = 5.4 Hz, 2H), 1.77 (p, *J* = 6.1 Hz, 2H), 1.64 (d, *J* = 5.2 Hz, 2H), 1.55 (d, *J* = 4.4 Hz, 4H) ppm.

1-(3-(2-Oxoazepan-1-yl)propyl)-3-phenethylthiourea (15): Off white solid 83 °C (water); ^{1}H-NMR: (500 MHz, $CDCl_3$) δ 7.45 (bs, 1H), 7.31–7.20 (m, 5H), 5.88 (s, 1H), 3.65–3.58 (m, 4H), 3.41 (t, *J* = 5.9 Hz, 2H), 3.34 (t, *J* = 5.0 Hz, 2H), 2.91 (t, *J* = 7.0 Hz, 2H), 2.54 (t, *J* = 5.9 Hz, 2H), 1.76–1.65 (m, 8H) ppm; ^{13}C-NMR: (125 MHz, $CDCl_3$) δ 172.6, 133.5, 124.0, 123.9, 121.9, 45.0, 40.4, 39.6, 36.1, 32.4, 30.3, 25.1, 23.6, 21.8, 18.7 ppm; HRMS (ESI/Q-TOF) m/z: $[M+H]^+$ Calcd. for $C_{18}H_{28}N_3OS$ 334.1947; found 334.1965.

1-(2-(1H-Indol-3-yl)ethyl)-3-(3-(2-oxoazepan-1-yl)propyl)thiourea (16): Yellow gum; ^{1}H-NMR: (500 MHz, $CDCl_3$) δ 8.63 (bs, 1H), 7.56 (d, *J* = 7.9 Hz, 1H), 7.33 (d, *J* = 8.1 Hz, 1H), 7.15 (t, *J* = 7.2 Hz, 1H), 7.07 (t, *J* = 7.8 Hz, 1H), 6.97 (d, *J* = 1.6 Hz, 1H), 6.13 (bs, 1H), 3.68 (bs, 2H), 3.51 (bs, 2H), 3.34–3.32 (m, 4H), 3.00 (t, *J* = 6.6 Hz, 2H), 2.48 (t, *J* = 5.5 Hz, 2H), 1.70–1.59 (m, 8H) ppm.

1-Benzyl-3-(2,6-dimethylphenyl)thiourea (26): White solid 106–107 °C (water); ^{1}H-NMR: (500 MHz, $CDCl_3$) δ 7.53 (bs, 1H), 7.33–7.26 (m, 5H), 7.20–7.12 (m, 3H), 5.64 (bs, 1H), 4.87 (d, *J* = 5.3 Hz, 2H), 2.28 (s, 6H) ppm.

1-Benzyl-3-(quinolin-3-yl)thiourea (27): Off white solid 147–148 °C (water); ^{1}H-NMR: (500 MHz, $CDCl_3$) δ 8.73 (d, *J* = 2.5 Hz, 1H), 8.34 (bs, 1H), 8.07 (d, *J* = 1.8 Hz, 1H), 7.99 (d, *J* = 8.5 Hz, 1H), 7.70–7.65 (m, 2H), 7.52 (d, *J* = 7.8 Hz, 1H), 7.32–7.25 (m, 5H), 6.69 (bs, 1H), 4.87 (*J* = 4.9 Hz, 2H) ppm; ^{13}C-NMR: (125 MHz, $CDCl_3$) δ 181.6, 172.5, 146.9, 131.6, 130.2, 129.8, 128.8, 127.9, 127.8, 127.7, 127.2, 123.8, 49.0 ppm; HRMS (ESI/Q-TOF) *m/z*: $[M + H]^+$ Calcd. for $C_{17}H_{16}N_3S$ 294.1059; found 294.1067.

1-Benzyl-3-phenethylthiourea (28): Compound have been characterized in ref.[6,7] The observed ^{1}H NMR spectrum was consistent with the previously reported data; Off white solid 101–102 °C (water; ref.[6] 118–120 °C, ethanol); ^{1}H-NMR: (500 MHz, $CDCl_3$, ref.[7]) δ 7.34–7.27 (m, 5H), 7.24–7.20 (m, 3H), 7.14–7.13 (m, 2H), 6.10 (bs, 1H), 5.74 (bs, 1H), 4.50 (m, 2H), 3.73 (m, 2H), 2.87 (t, *J* = 6.9 Hz, 2H) ppm; HRMS (ESI/Q-TOF) *m/z*: $[M + H]^+$ Calcd. for $C_{16}H_{19}N_2S$ 271.1263; found 271.1265.

1-Benzyl-3-cyclohexylthiourea (29): Compound have been characterized in ref.[8,9] The observed ^{1}H NMR spectrum was consistent with the previously reported data; Beige solid, m.p. 108–109 °C (water; ref.[8] 89–90 °C); ^{1}H-NMR: (500 MHz, $CDCl_3$, ref.[9]) δ 7.34–7.30 (m, 5H), 6.16 (bs, 1H), 5.79 (bs, 1H), 4.62 (bs, 2H), 3.85 (bs, 1H), 1.95–1.93 (m, 2H), 1.65–1.56 (m, 3H), 1.34–1.13 (m, 5H) ppm.

1-Benzyl-3-(*tert*-butyl)thiourea (30): Compound have been characterized in ref.[10] The observed ^{1}H NMR spectrum was consistent with the previously reported data; White solid, m.p. 93–94 °C (water; ref.[10] 94 °C); ^{1}H-NMR: (500 MHz, $CDCl_3$, mixture of E/Z isomers, ref[10]) δ 7.38–7.30 (m, 5H), 6.01 (bs, 1H), 5.86 (bs, 1H), 4.78–4.77 (m, 2H), 1.39 (s, 9H) ppm.

(*S*)-1-(2,6-Dimethylphenyl)-3-(1-phenylethyl)thiourea (31): White solid, m.p. 117–118 °C (water); ^{1}H-NMR: (500 MHz, $CDCl_3$, mixture of E/Z isomers) δ 7.56 (bs, 1H), 7.30–7.11 (m, 8H), 5.75 (bs, 1H), 5.48 (bs, 1H), 2.24 ((two signals: 2.30 (s), 2.17 (s)) 3H), 1.48 ((two signals: 1.48 (s), 1.47 (s)) 3H) ppm.

***N*-(2,6-Dimethylphenyl)morpholine-4-carbothioamide (32)**: Compound have been characterized in ref.[11] The observed ^{1}H NMR spectrum was consistent with the previously reported data (although the solvent was DMSO-*d6*); White solid, m.p. 163–164 °C (water; ref.[11] 138–140 °C); ^{1}H-NMR: (500 MHz, $CDCl_3$, ref.[11]) δ 7.15–7.08 (m, 3H), 6.84 (bs, 1H), 3.84 (t, *J* = 4.4 Hz, 2H), 3.73 (t, *J* = 5.1 Hz, 2H), 2.25 (s, 6H) ppm.

1-(2,6-Dimethylphenyl)-3-phenylthiourea (33): Compound have been characterized in ref.[12,13] The observed ^{1}H NMR spectrum was consistent with the previously reported data; White solid, m.p. 170–171 °C (water; ref.[12] 203–205 °C); ^{1}H-NMR: (500 MHz, DMSO-d_6, ref.[13]) δ 9.87 (bs, 1H), 8.98 (bs, 1H), 7.52–7.09 (m, 8H), 2.22 (s, 6H) ppm.

1-(2,6-Dimethylphenyl)-3-(*p*-tolyl)thiourea (34): White solid, m.p. 156–157 °C (water); ^{1}H-NMR: (500 MHz, DMSO-d_6) δ 9.75 (bs, 1H), 8.84 (bs, 1H), 7.34–7.08 (m, 7H), 2.28 (s, 3h), 2.21 (s, 6H) ppm; ^{13}C-NMR: (125 MHz, DMSO-d_6) δ 180.2, 137.2 (bs), 136.8, 136.3, 133.7, 129.2 (bs), 128.8, 128.1, 127.6 (bs), 126.7 (bs), 123.9, 123.6 (bs), 20.5, 18.1 ppm; HRMS (ESI/Q-TOF) *m/z*: $[M + H]^+$ Calcd. for $C_{16}H_{19}N_2S$ 271.1263; found 217.1268.

1-(4-Chlorophenyl)-3-(2,6-dimethylphenyl)thiourea (35): Off white solid 136–137 °C (water); ^{1}H-NMR: (500 MHz, DMSO-d_6) δ 9.93 (bs, 1H), 9.02 (bs, 1H), 7.55–7.27 (m, 4H), 7.09–7.06 (m, 3H), 2.20 (s, 6H) ppm; ^{13}C-NMR: (125 MHz, DMSO-d_6) δ 180.3, 138.3, 136.3, 128.5 (bs), 128.3, 127.7 (bs), 126.8 (bs), 125.3, 124.7 (bs), 18.0 ppm; HRMS (ESI/Q-TOF) *m/z*: $[M + H]^+$ Calcd. for $C_{15}H_{16}ClN_2S$ 291.0717; found 291.0725.

1-(4-Bromophenyl)-3-(2,6-dimethylphenyl)thiourea (36): Off white solid 128–129 °C (water); ^{1}H-NMR: (500 MHz, DMSO-d_6) δ 9.91 (bs, 1H), 9.04 (bs, 1H), 7.52–7.45 (m, 5H), 7.09 (m, 3H), 2.21 (s, 3H) ppm.

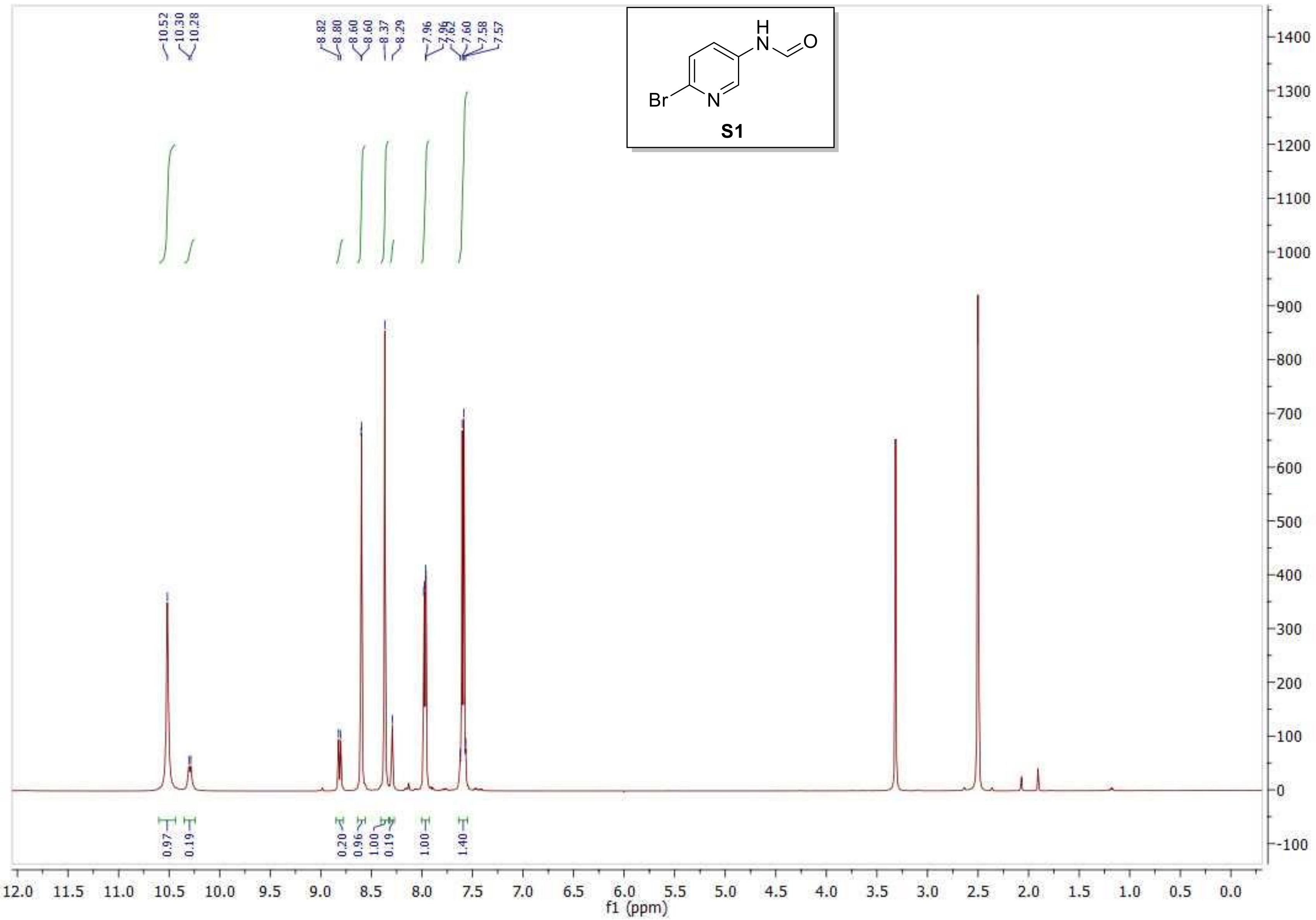
10.52
10.30
10.28
8.82
8.80
8.60
8.60
8.37
8.29
7.96
7.96
7.62
7.60
7.58
7.57
H
N
O
Br
N
S1
0.97
0.19
0.20
0.96
1.00
0.19
1.00
1.40
12.0 11.5 11.0 10.5 10.0 9.5 9.0 8.5 8.0 7.5 7.0 6.5 6.0 5.5 5.0 4.5 4.0 3.5 3.0 2.5 2.0 1.5 1.0 0.5 0.0
f1 (ppm)
1400 1300 1200 1100 1000 900 800 700 600 500 400 300 200 100 0 -100

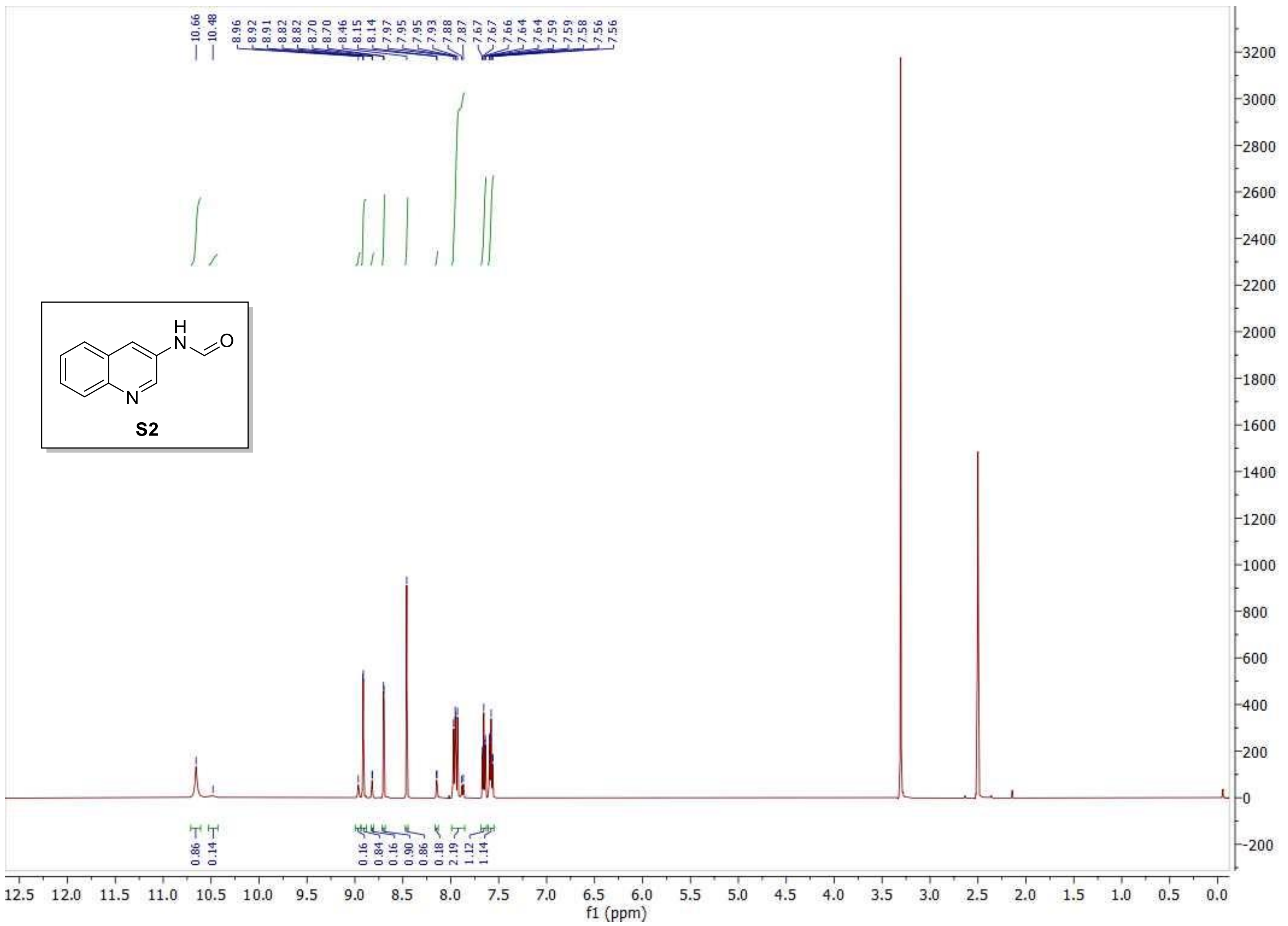
10.66
10.48
8.96
8.92
8.91
8.82
8.82
8.70
8.70
8.46
8.15
8.14
7.97
7.95
7.95
7.93
7.88
7.87
7.67
7.67
7.66
7.64
7.64
7.59
7.59
7.58
7.56
7.56
H
N
O
N
S2
0.86
0.14
0.16
0.84
0.16
0.90
0.86
0.18
2.19
1.12
1.14
f1 (ppm)

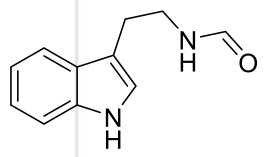

S3

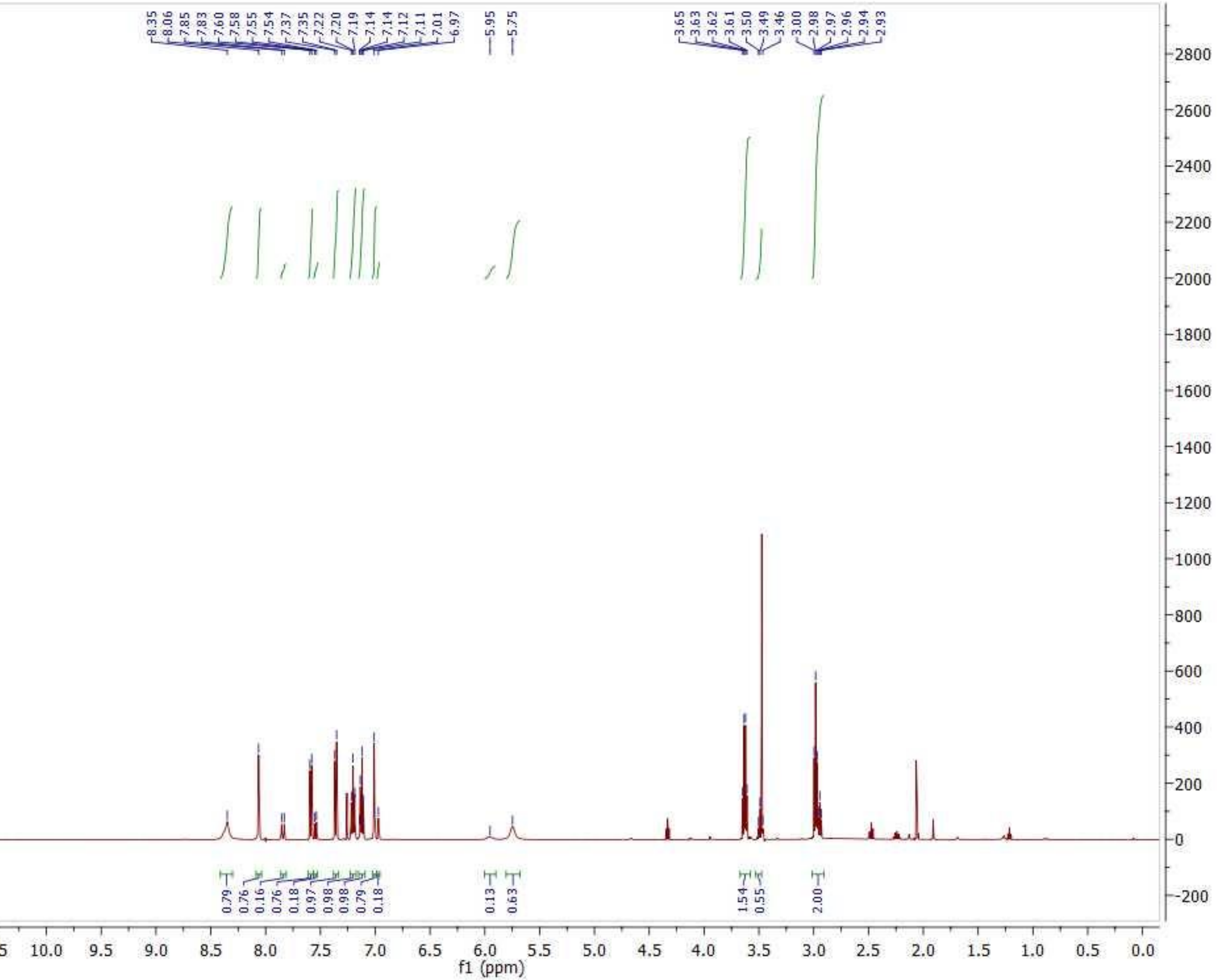
8.35 8.06 7.85 7.83 7.60 7.58 7.55 7.54 7.37 7.35 7.22 7.20 7.19 7.14 7.12 7.11 7.01 6.97
5.95
5.75
3.65 3.63 3.62 3.61 3.50 3.49 3.46 3.00 2.98 2.97 2.96 2.94 2.93
0.79 0.76 0.16 0.76 0.18 0.97 0.98 0.98 0.79 0.18
0.13 0.63
1.54 0.55
2.00
12.0 11.5 11.0 10.5 10.0 9.5 9.0 8.5 8.0 7.5 7.0 6.5 6.0 5.5 5.0 4.5 4.0 3.5 3.0 2.5 2.0 1.5 1.0 0.5 0.0
f1 (ppm)
2800 2600 2400 2200 2000 1800 1600 1400 1200 1000 800 600 400 200 0 -200

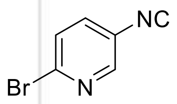

5

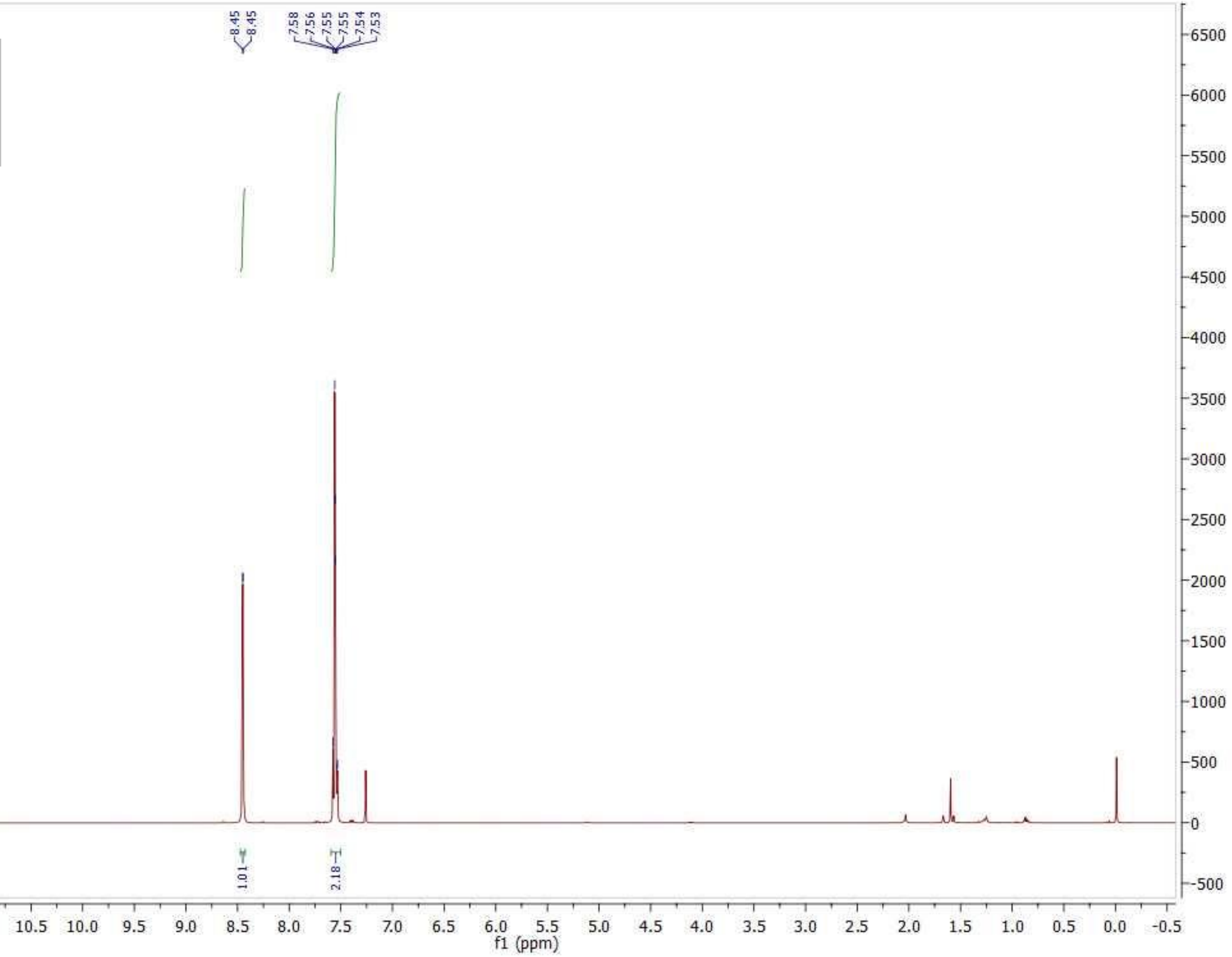

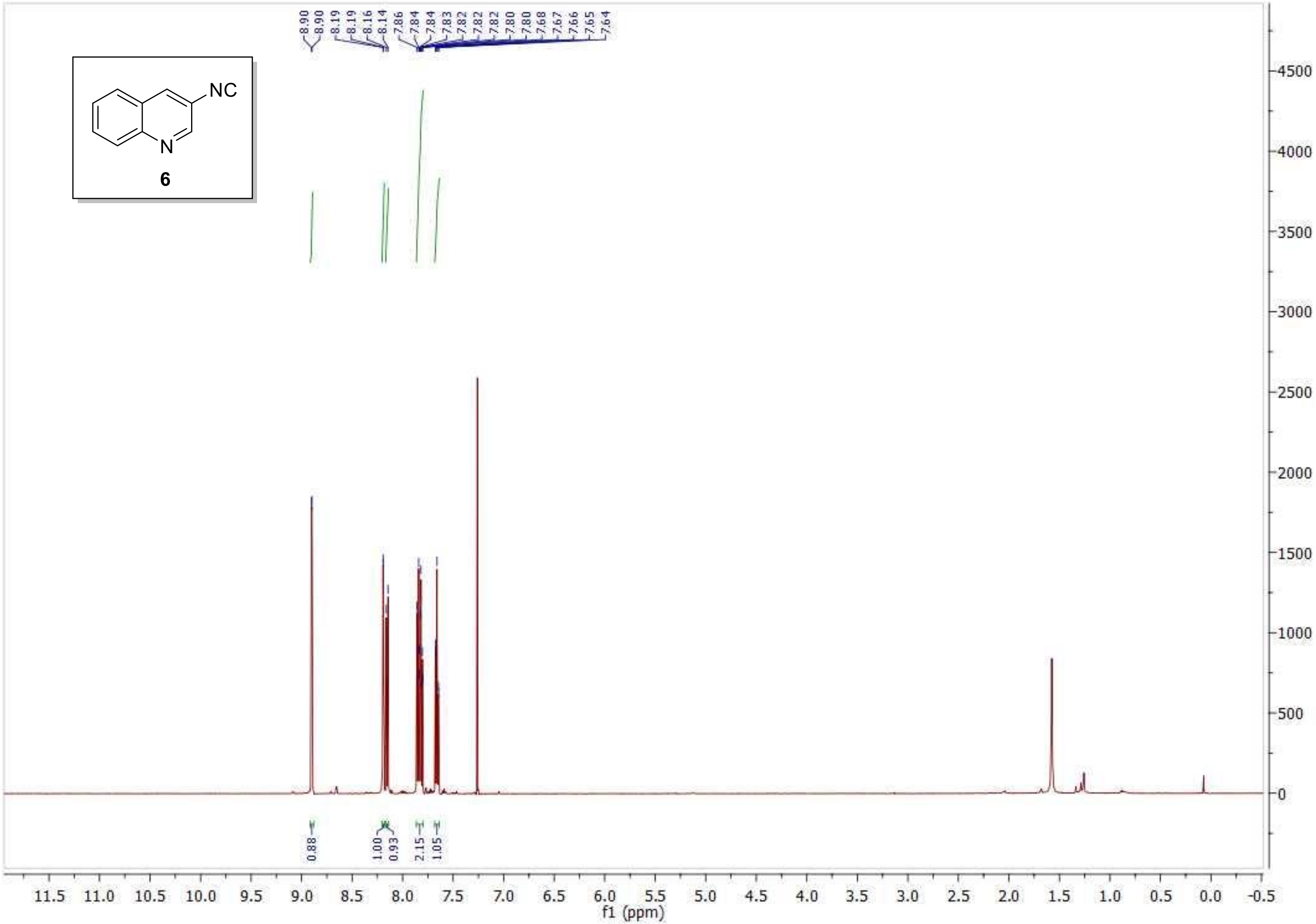
NC
N
6
8.90
8.90
8.19
8.19
8.16
8.14
7.86
7.84
7.84
7.83
7.82
7.82
7.82
7.80
7.80
7.68
7.67
7.66
7.65
7.64
0.88
1.00
0.93
2.15
1.05
4500
4000
3500
3000
2500
2000
1500
1000
500
0
11.5
11.0
10.5
10.0
9.5
9.0
8.5
8.0
7.5
7.0
6.5
6.0
5.5
5.0
4.5
4.0
3.5
3.0
2.5
2.0
1.5
1.0
0.5
0.0
-0.5
f1 (ppm)

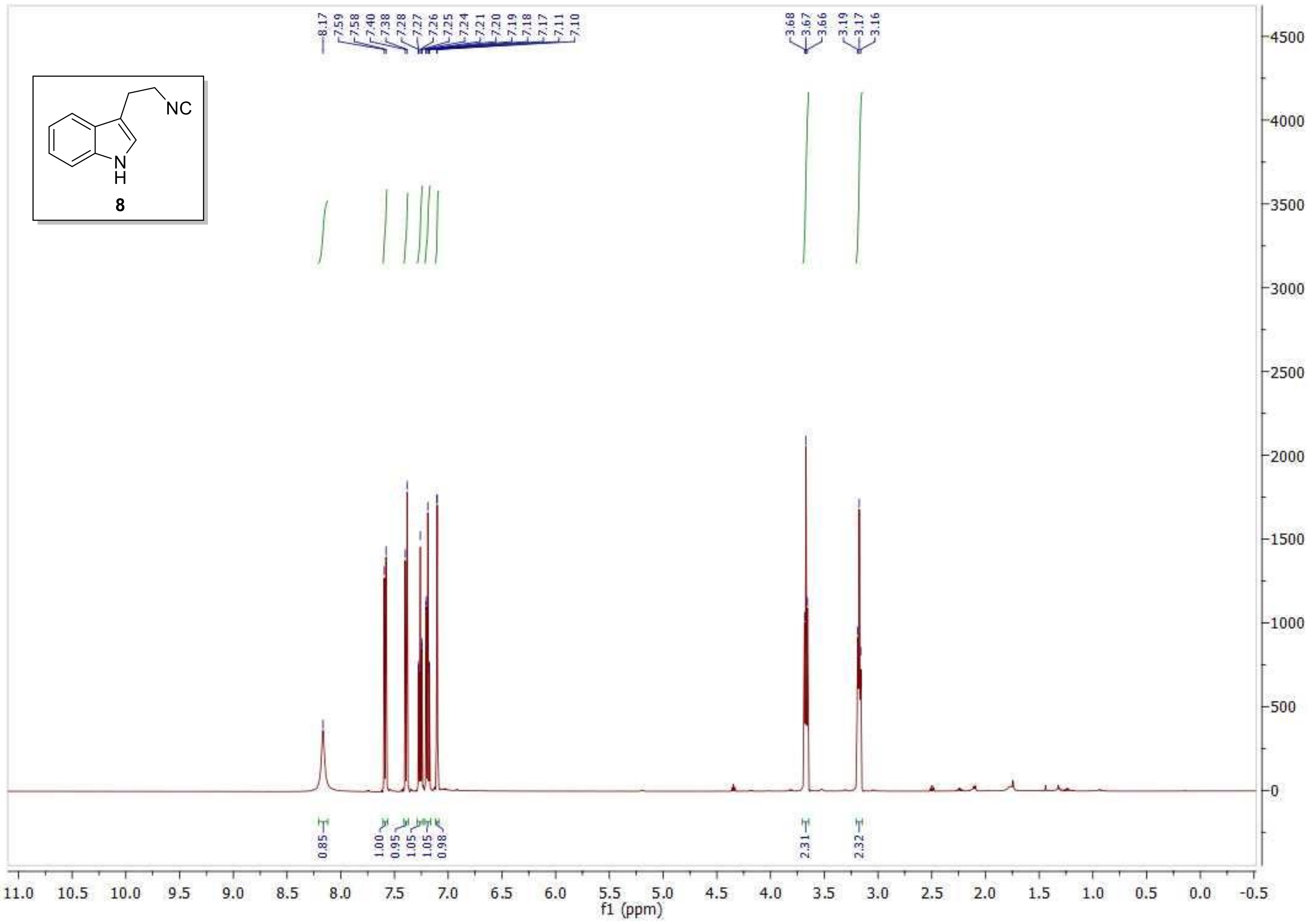

8.17
7.59
7.58
7.40
7.38
7.28
7.27
7.26
7.25
7.24
7.21
7.20
7.19
7.18
7.17
7.11
7.10
3.68
3.67
3.66
3.19
3.17
3.16
NC
N
H
8
0.85
1.00
0.95
1.05
1.05
0.98
2.31
2.32
f1 (ppm)

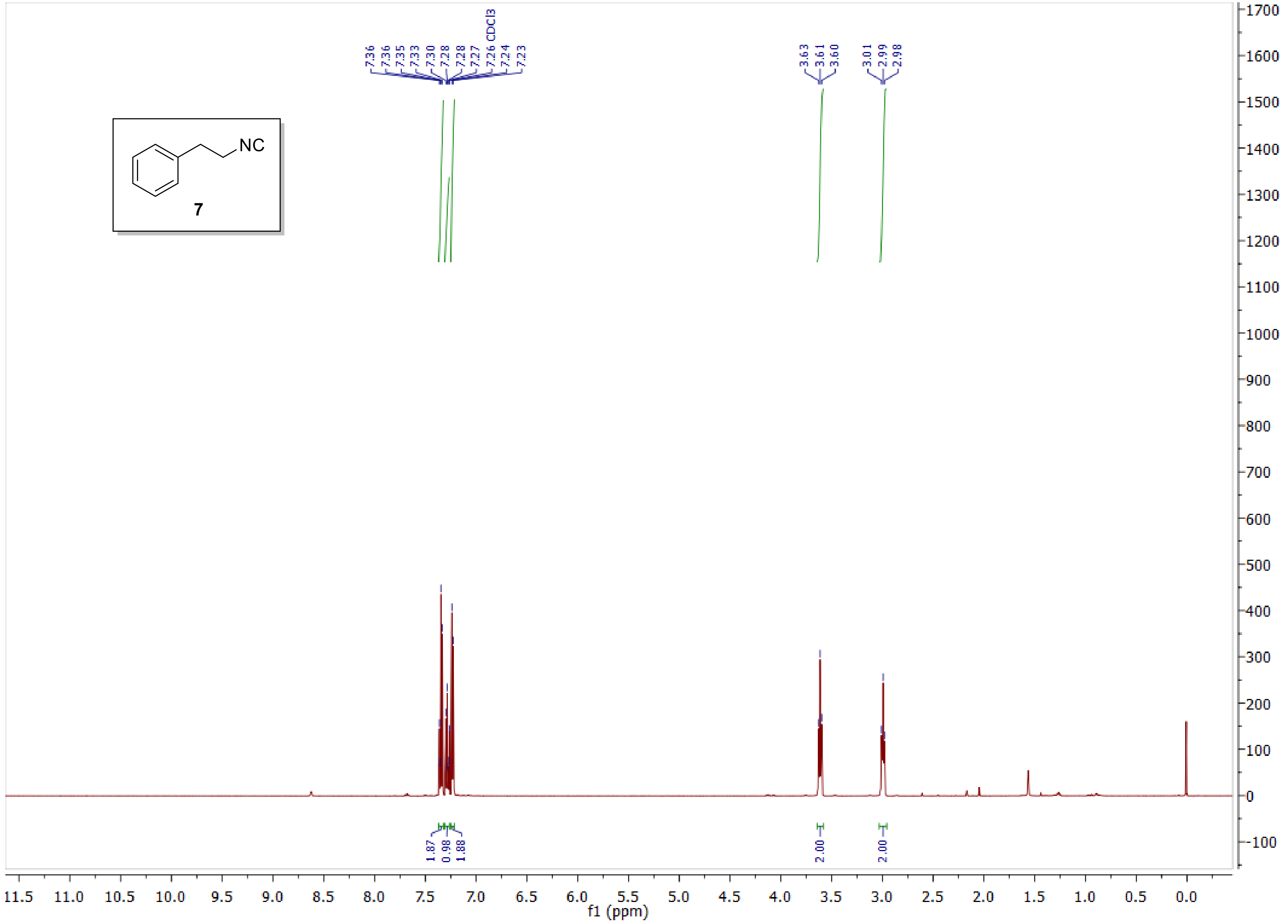
NC
7
7.36
7.36
7.35
7.33
7.30
7.28
7.28
7.27
7.26 CDCl3
7.24
7.23
3.63
3.61
3.60
3.01
2.99
2.98
1.87
0.98
1.88
2.00
2.00
f1 (ppm)

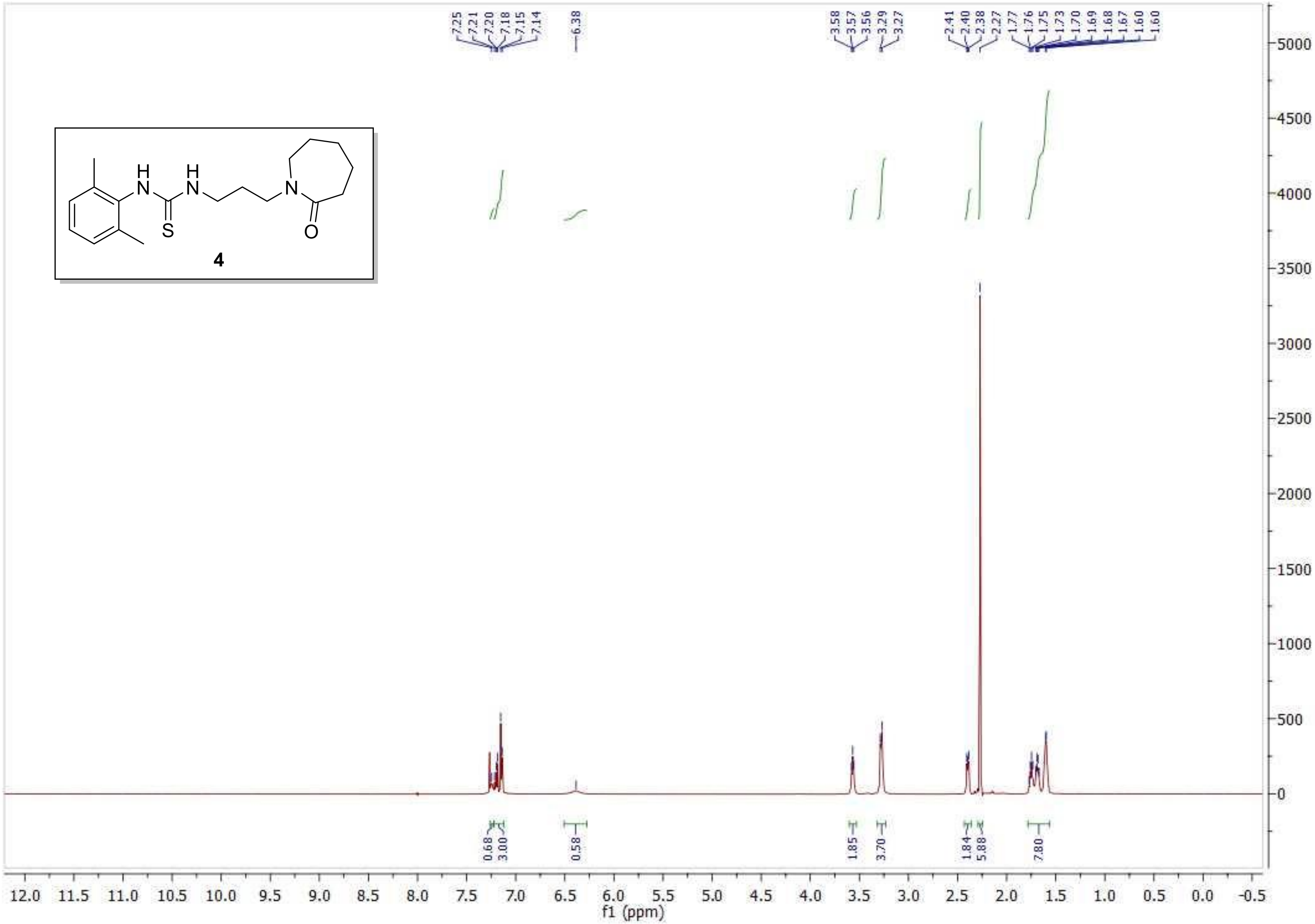

7.25
7.21
7.20
7.18
7.15
7.14
6.38
3.58
3.57
3.56
3.29
3.27
2.41
2.40
2.38
2.27
1.77
1.76
1.75
1.73
1.70
1.69
1.68
1.67
1.60
1.60
H
N
S
O
4
0.68
3.00
0.58
1.85
3.70
1.84
5.88
7.80
12.0 11.5 11.0 10.5 10.0 9.5 9.0 8.5 8.0 7.5 7.0 6.5 6.0 5.5 5.0 4.5 4.0 3.5 3.0 2.5 2.0 1.5 1.0 0.5 0.0 -0.5
f1 (ppm)
5000 4500 4000 3500 3000 2500 2000 1500 1000 500 0

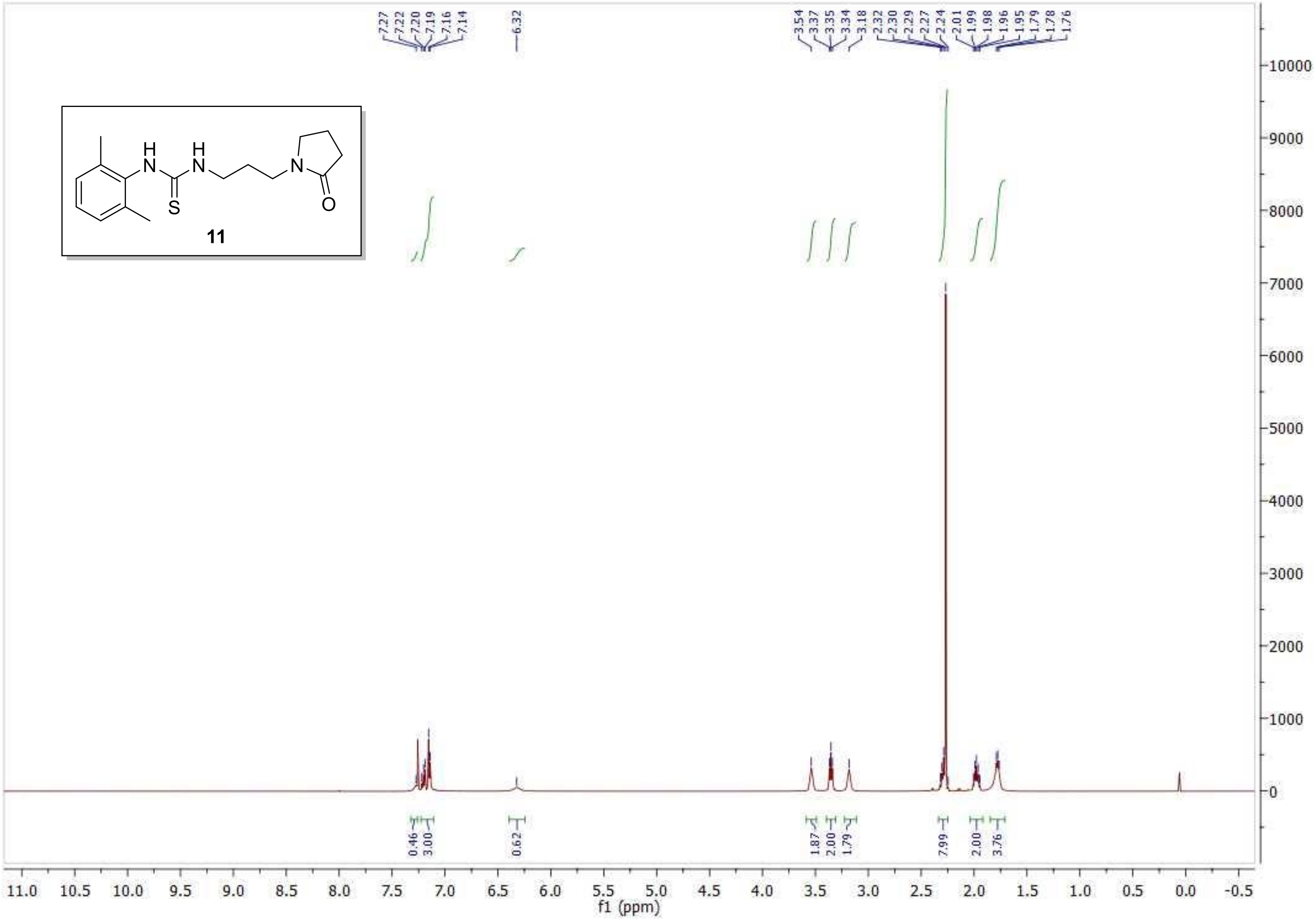

7.27
7.22
7.20
7.19
7.16
7.14
6.32
3.54
3.37
3.35
3.34
3.18
2.32
2.30
2.29
2.27
2.24
2.01
1.99
1.98
1.96
1.95
1.79
1.78
1.76
11
0.46
3.00
0.62
1.87
2.00
1.79
7.99
2.00
3.76
f1 (ppm)

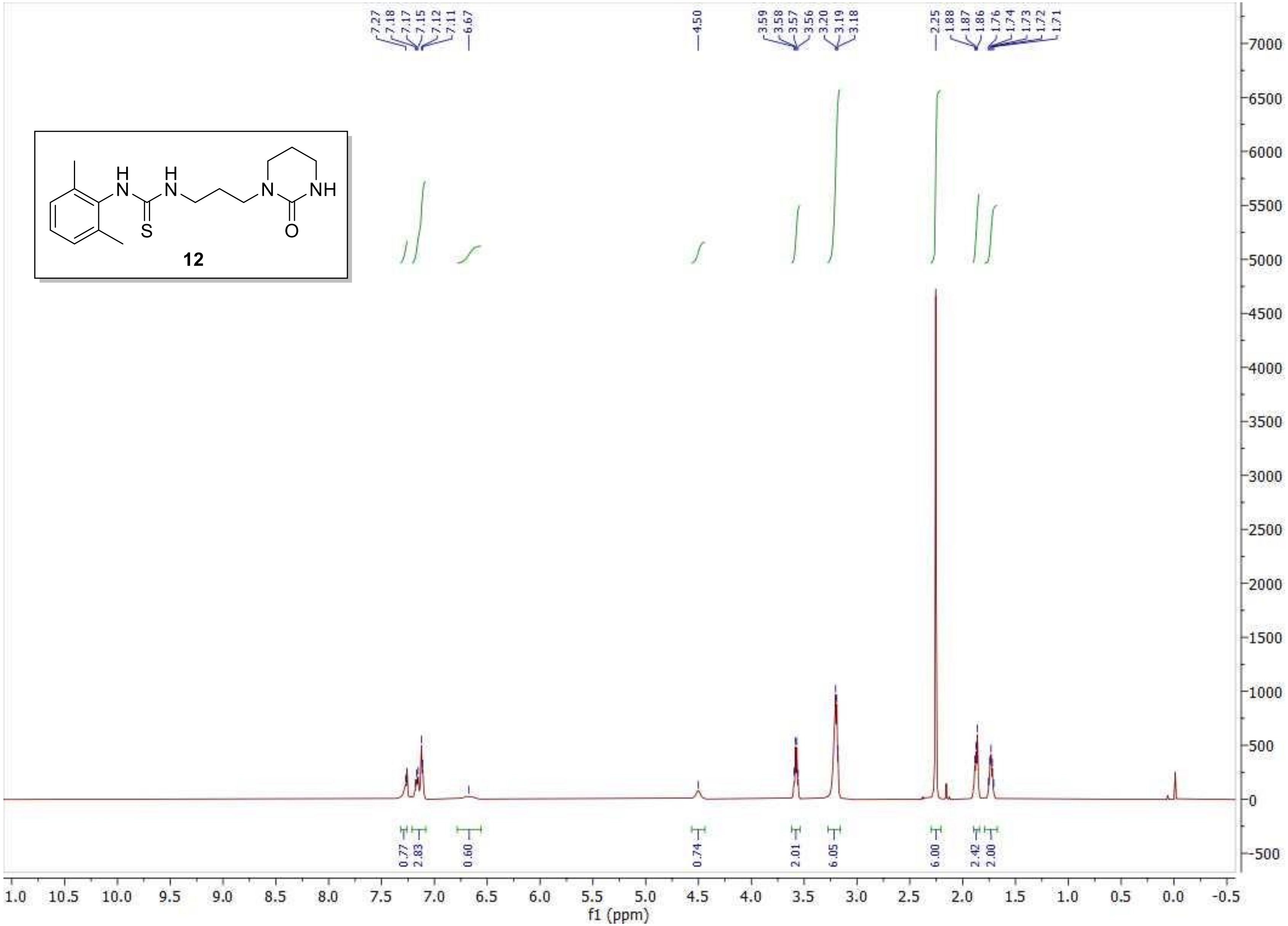

7.27
7.18
7.17
7.15
7.12
7.11
6.67
4.50
3.59
3.58
3.57
3.56
3.20
3.19
3.18
2.25
1.88
1.87
1.86
1.76
1.74
1.73
1.72
1.71
0.77
2.83
0.60
0.74
2.01
6.05
6.00
2.42
2.00
12
f1 (ppm)

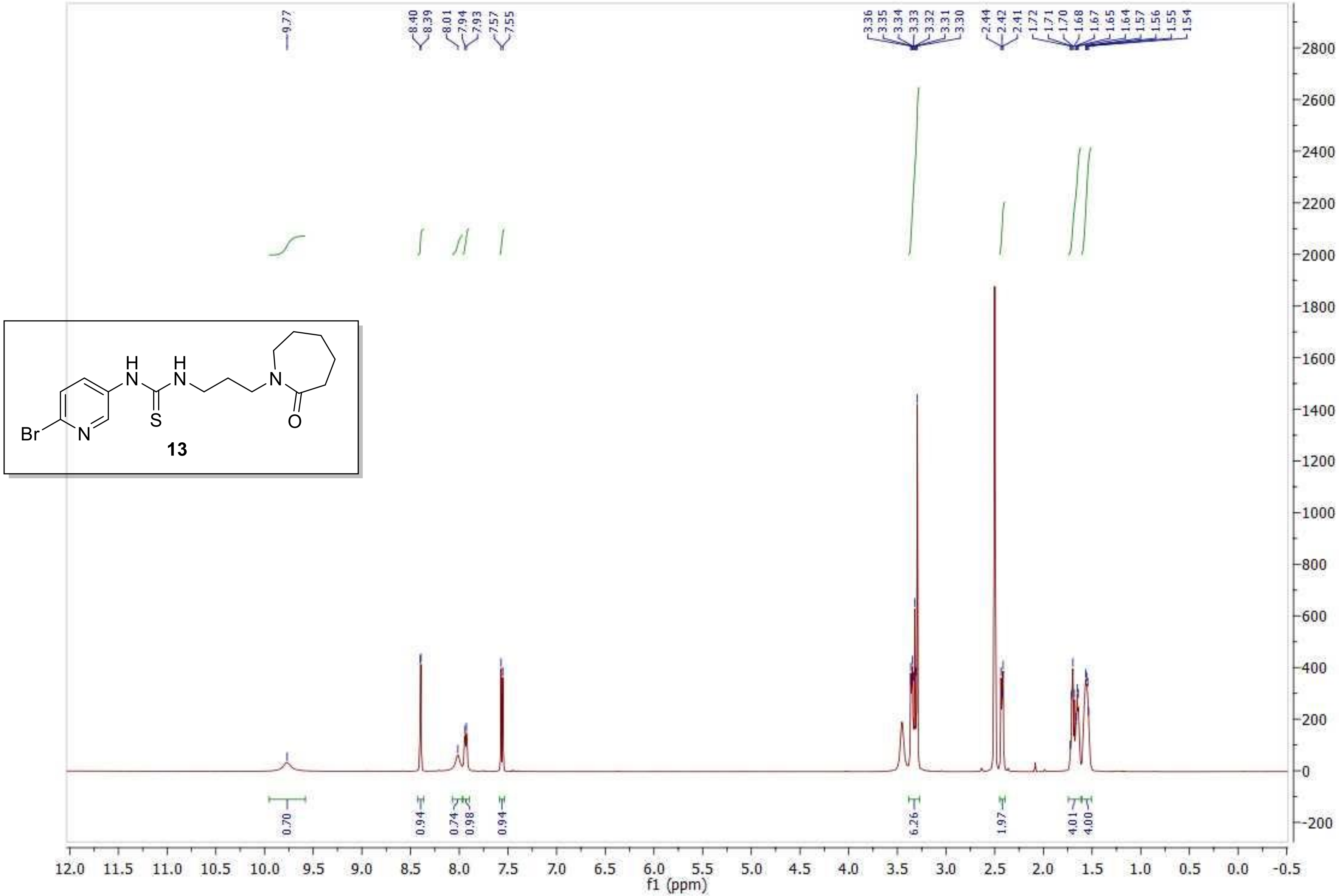

9.77
8.40 8.39 8.01 7.94 7.93 7.57 7.55
3.36 3.35 3.34 3.33 3.32 3.31 3.30
2.44 2.42 2.41
1.72 1.71 1.70 1.68 1.67 1.65 1.64 1.57 1.56 1.55 1.54
0.70 0.94 0.74 0.98 0.94 6.26 1.97 4.01 4.00
Br N H N H N S N O
13
f1 (ppm)
12.0 11.5 11.0 10.5 10.0 9.5 9.0 8.5 8.0 7.5 7.0 6.5 6.0 5.5 5.0 4.5 4.0 3.5 3.0 2.5 2.0 1.5 1.0 0.5 0.0 -0.5
2800 2600 2400 2200 2000 1800 1600 1400 1200 1000 800 600 400 200 0 -200

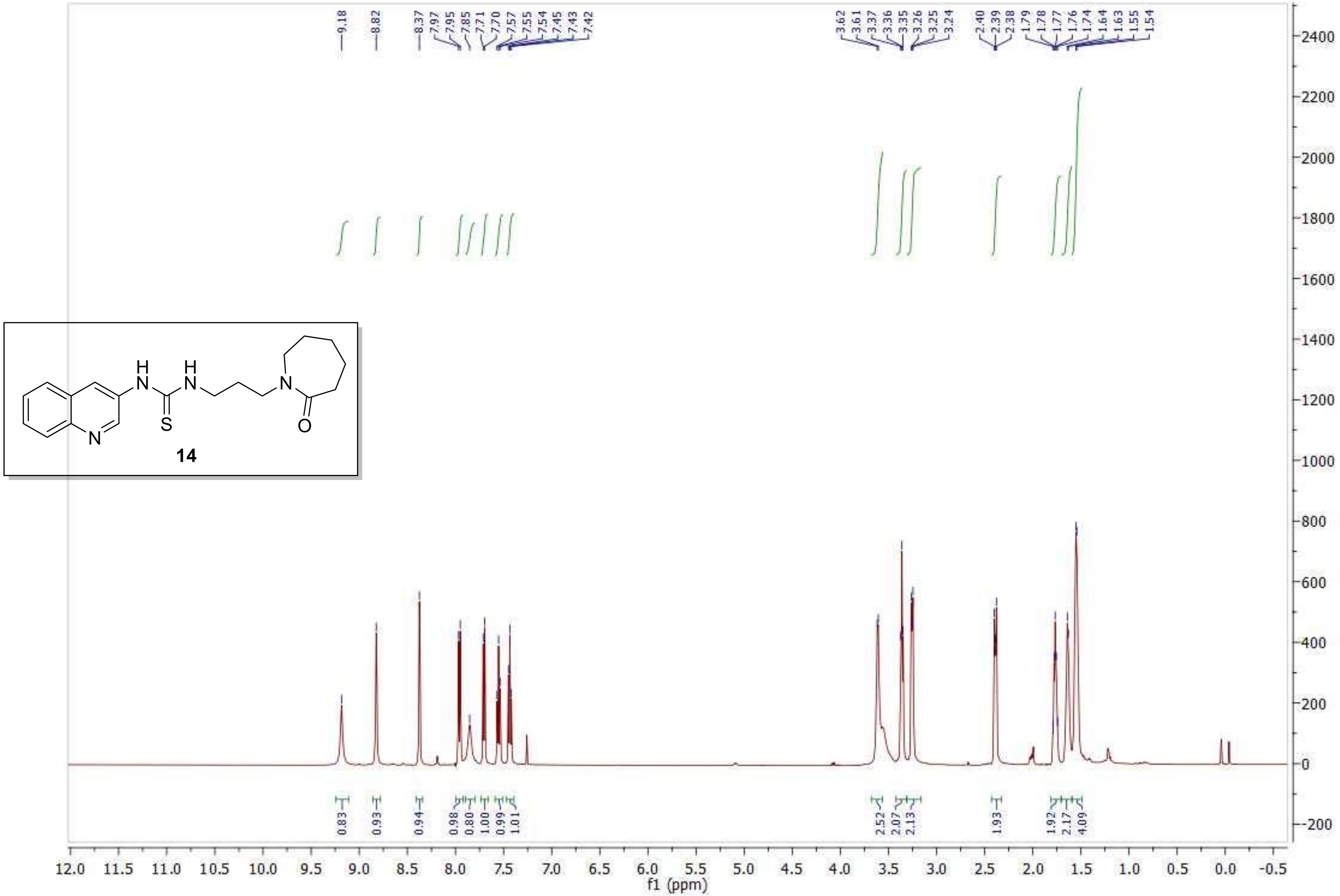
9.18
8.82
8.37
7.97
7.95
7.85
7.71
7.70
7.57
7.55
7.54
7.45
7.43
7.42
3.62
3.61
3.37
3.36
3.35
3.26
3.25
3.24
2.40
2.39
2.38
1.79
1.78
1.77
1.76
1.74
1.64
1.63
1.55
1.54
0.83
0.93
0.94
0.98
0.80
1.00
0.99
1.01
2.52
2.07
2.13
1.93
1.92
2.17
4.09
f1 (ppm)

14

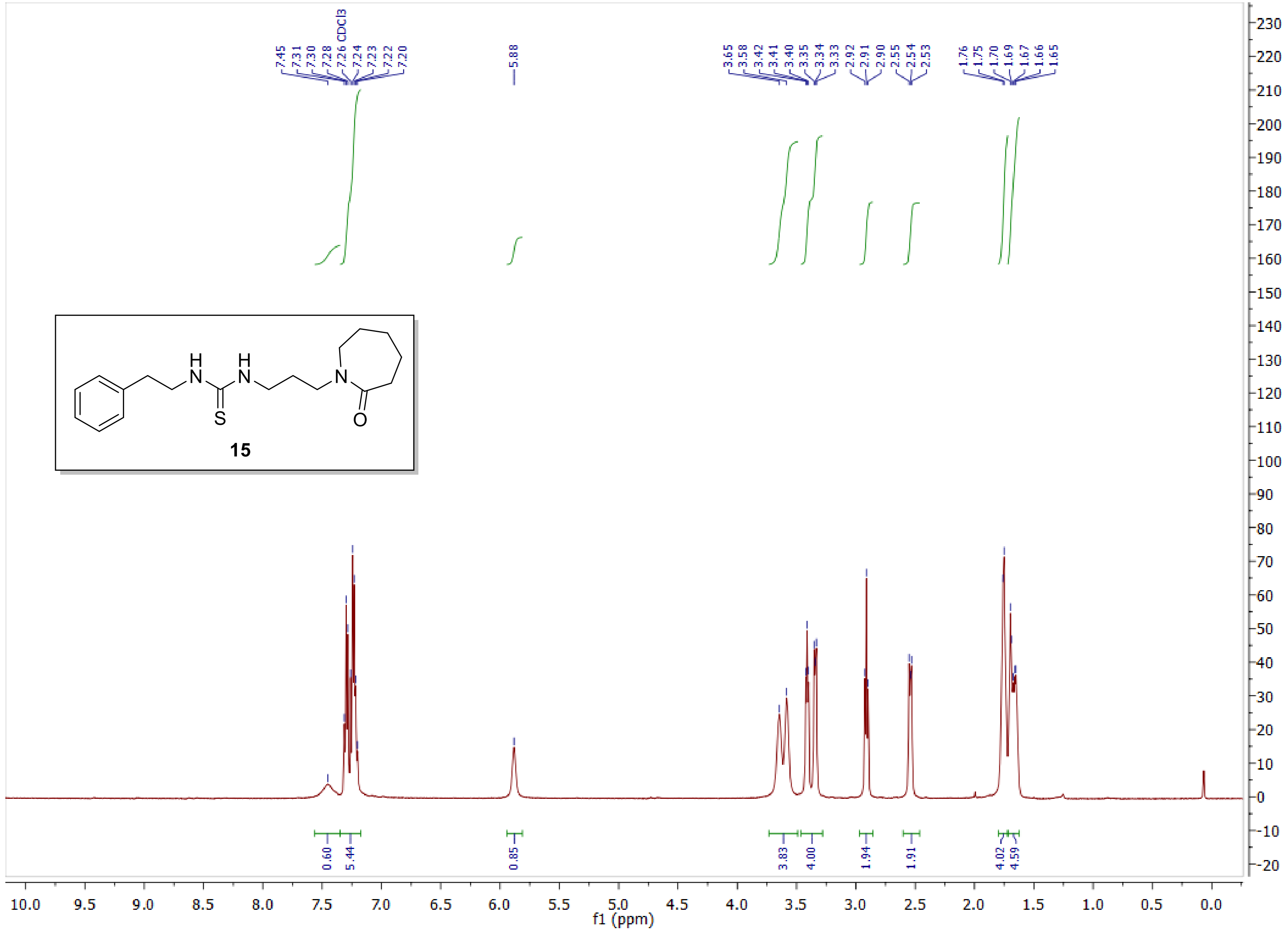
7.45
7.31
7.30
7.28
7.26 CDCl3
7.24
7.23
7.22
7.20
5.88
3.65
3.58
3.42
3.41
3.40
3.35
3.34
3.33
2.92
2.91
2.90
2.55
2.54
2.53
1.76
1.75
1.70
1.69
1.67
1.66
1.65
15
0.60
5.44
0.85
3.83
4.00
1.94
1.91
4.02
4.59
f1 (ppm)

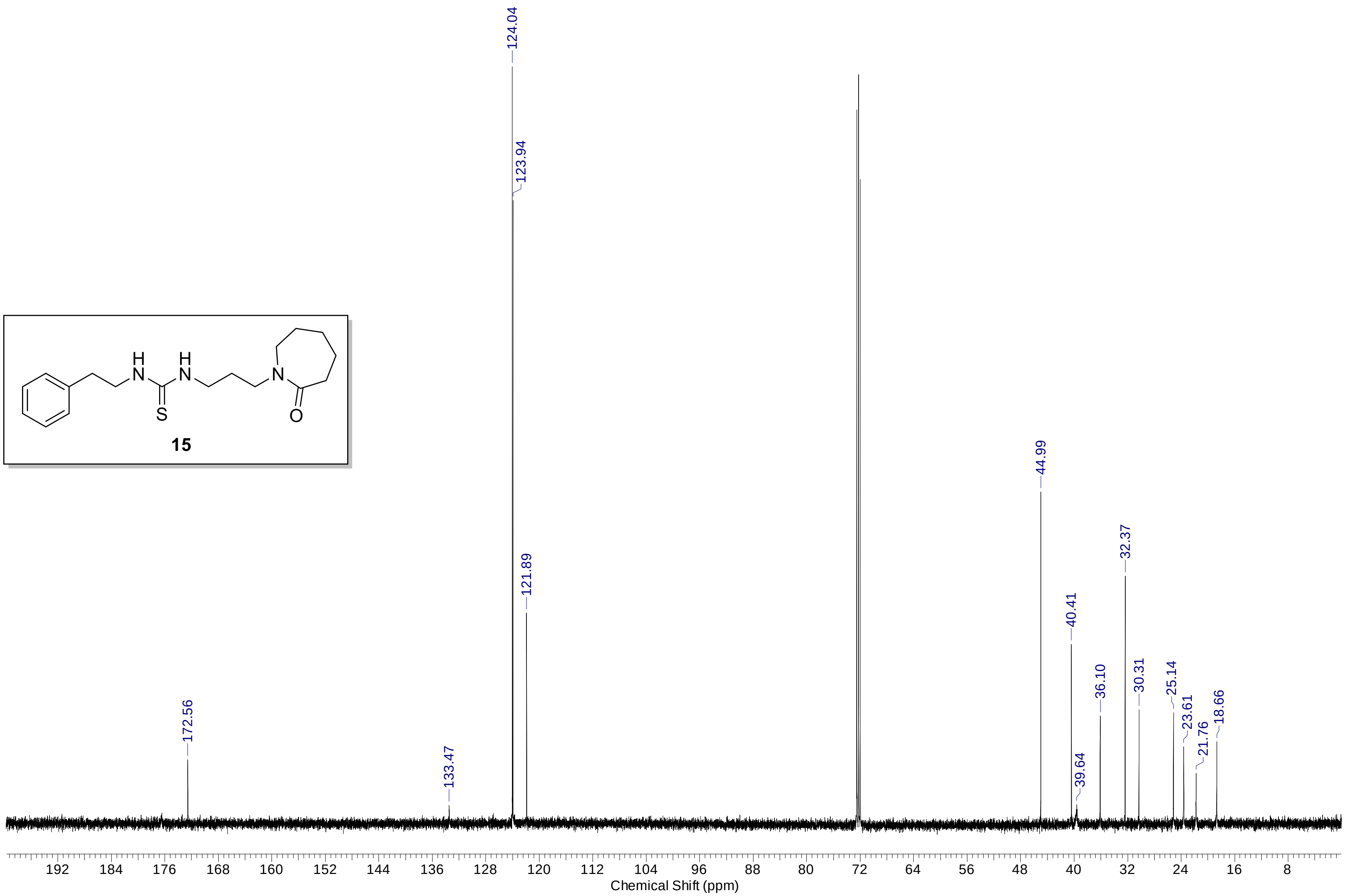
124.04
123.94
121.89
172.56
133.47
44.99
40.41
39.64
36.10
32.37
30.31
25.14
23.61
21.76
18.66
H
N
S
O
15
192 184 176 168 160 152 144 136 128 120 112 104 96 88 80 72 64 56 48 40 32 24 16 8
Chemical Shift (ppm)

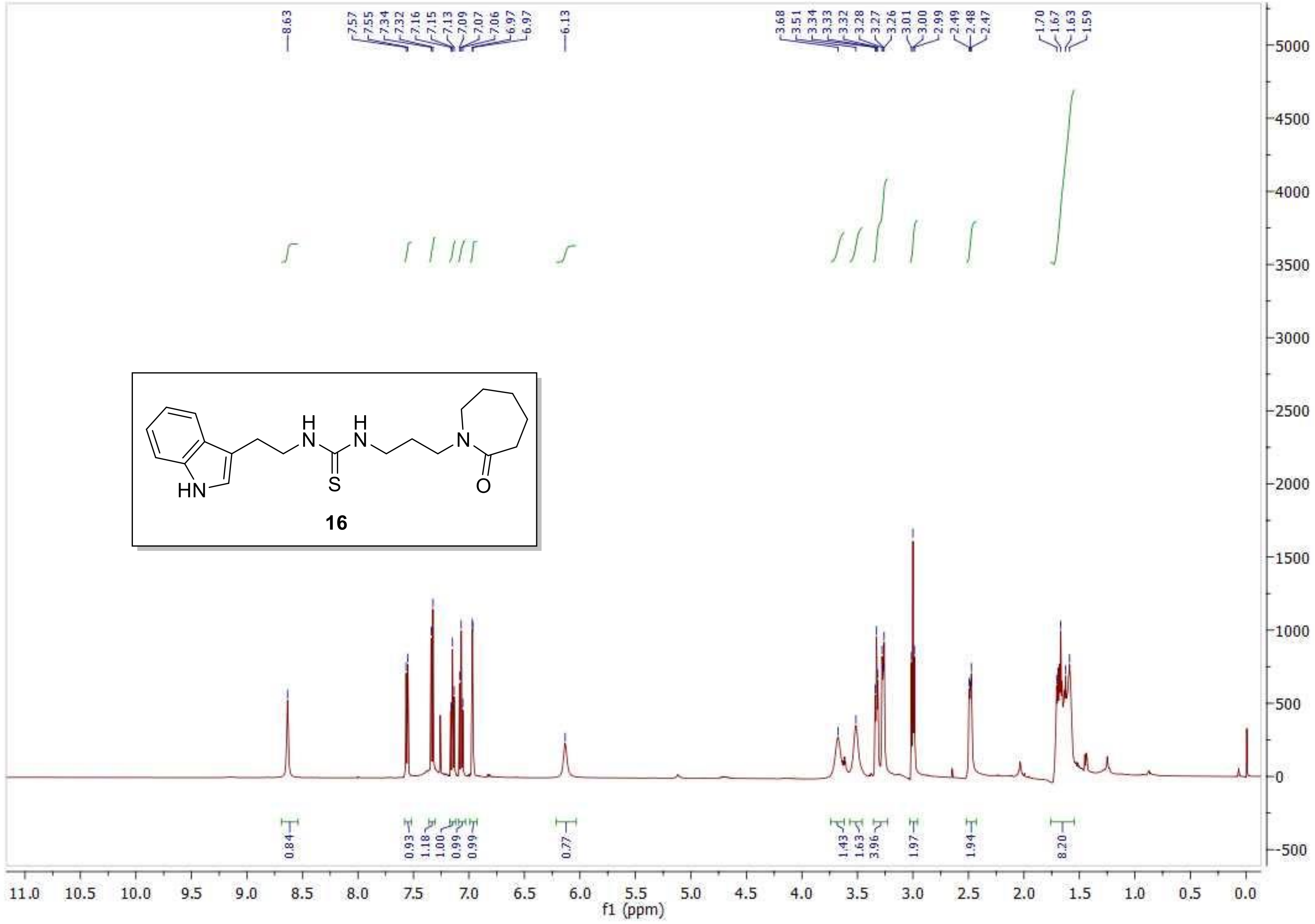
8.63
7.57
7.55
7.34
7.32
7.16
7.15
7.13
7.09
7.07
7.06
6.97
6.97
6.13
3.68
3.51
3.34
3.33
3.32
3.28
3.27
3.26
3.01
3.00
2.99
2.49
2.48
2.47
1.70
1.67
1.63
1.59
HN
H
N
H
N
S
N
O
16
0.84
0.93
1.18
1.00
0.99
0.99
0.99
0.77
1.43
1.63
3.96
1.97
1.94
8.20
11.0
10.5
10.0
9.5
9.0
8.5
8.0
7.5
7.0
6.5
6.0
5.5
5.0
4.5
4.0
3.5
3.0
2.5
2.0
1.5
1.0
0.5
0.0
f1 (ppm)
5000
4500
4000
3500
3000
2500
2000
1500
1000
500
0
-500

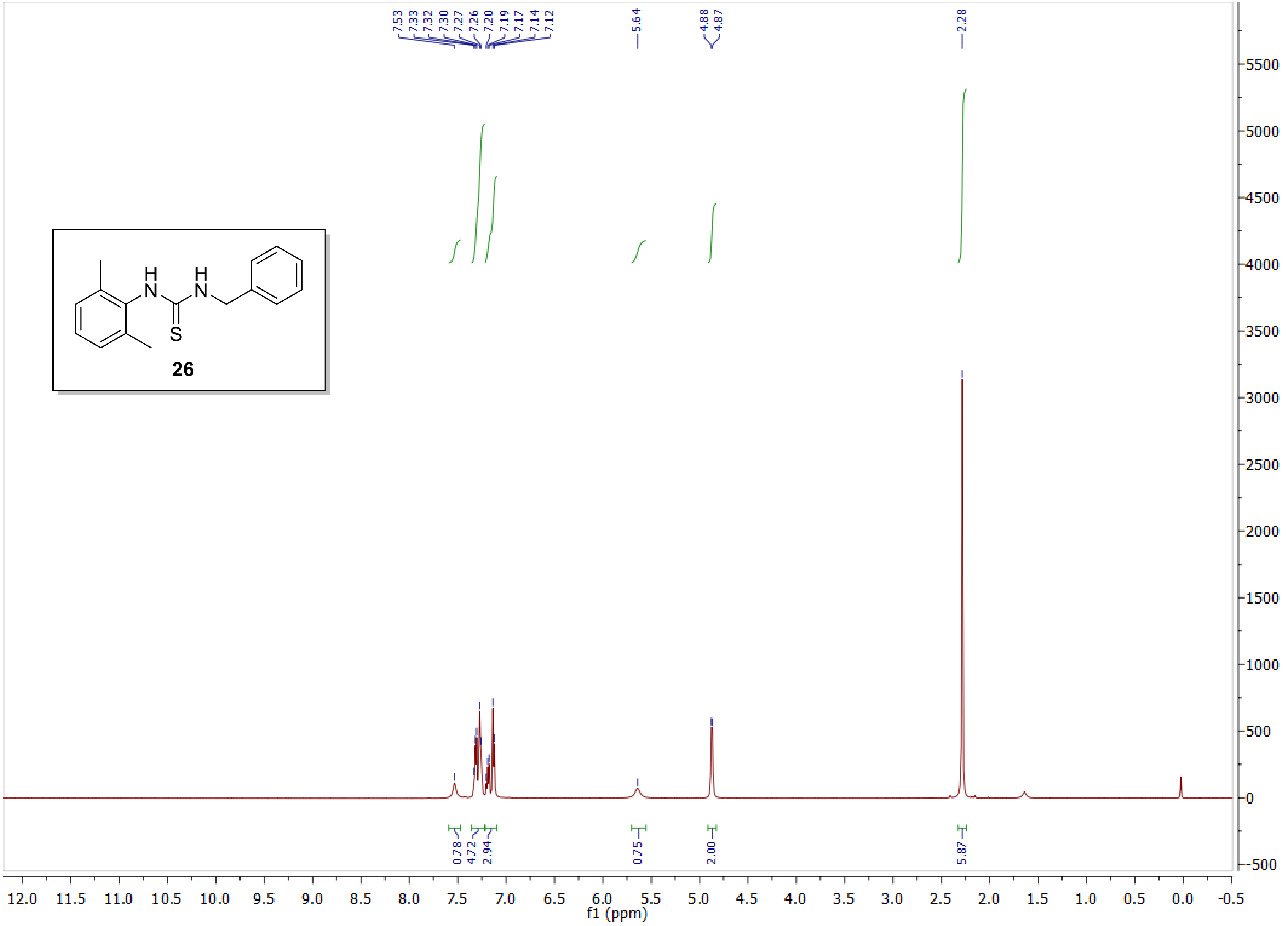
7.53
7.33
7.32
7.30
7.27
7.26
7.20
7.19
7.17
7.14
7.12
5.64
4.88
4.87
2.28
26
0.78
4.72
2.94
0.75
2.00
5.87
f1 (ppm)

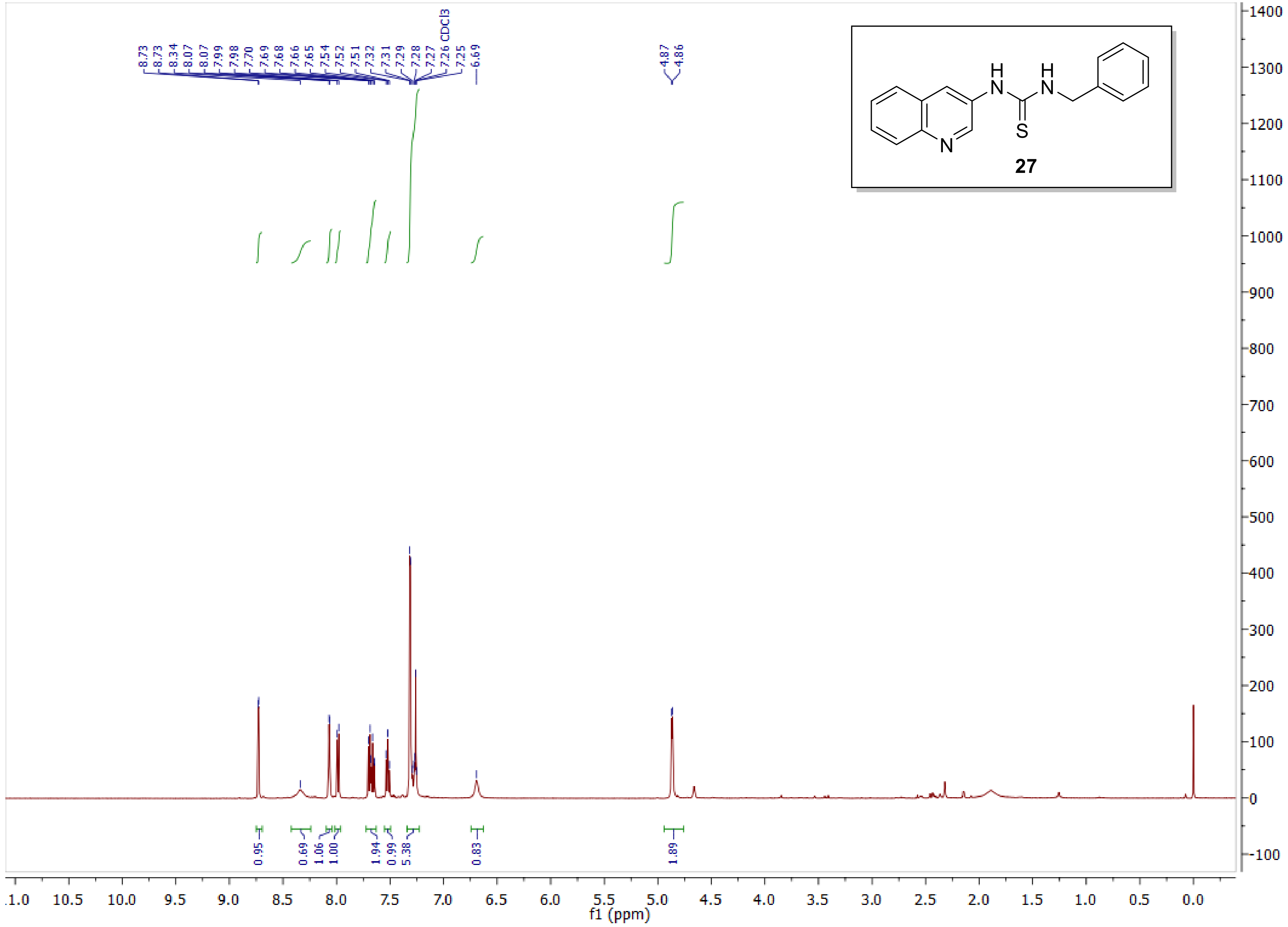

8.73
8.73
8.34
8.07
8.07
7.99
7.98
7.70
7.69
7.68
7.66
7.65
7.54
7.52
7.51
7.32
7.31
7.29
7.28
7.27
7.26 CDCl3
7.25
6.69
4.87
4.86
0.95
0.69
1.06
1.00
1.94
0.99
5.38
0.83
1.89
f1 (ppm)
27

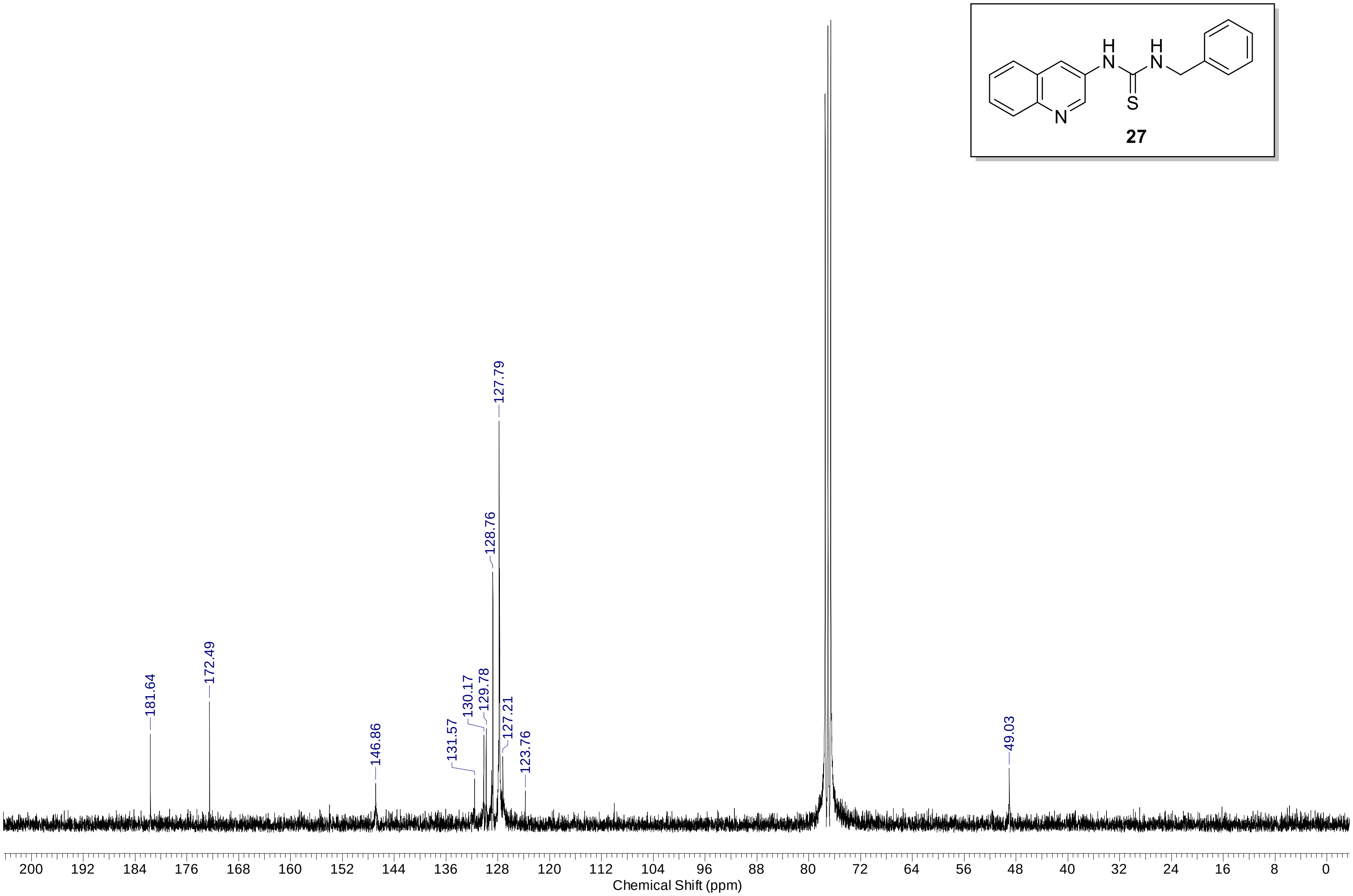

27
181.64
172.49
146.86
131.57
130.17
129.78
128.76
127.79
127.21
123.76
49.03
200 192 184 176 168 160 152 144 136 128 120 112 104 96 88 80 72 64 56 48 40 32 24 16 8 0
Chemical Shift (ppm)

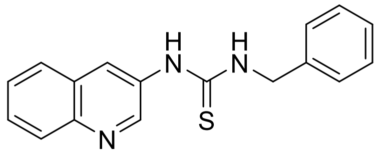

H
N
H
N
S
N

27

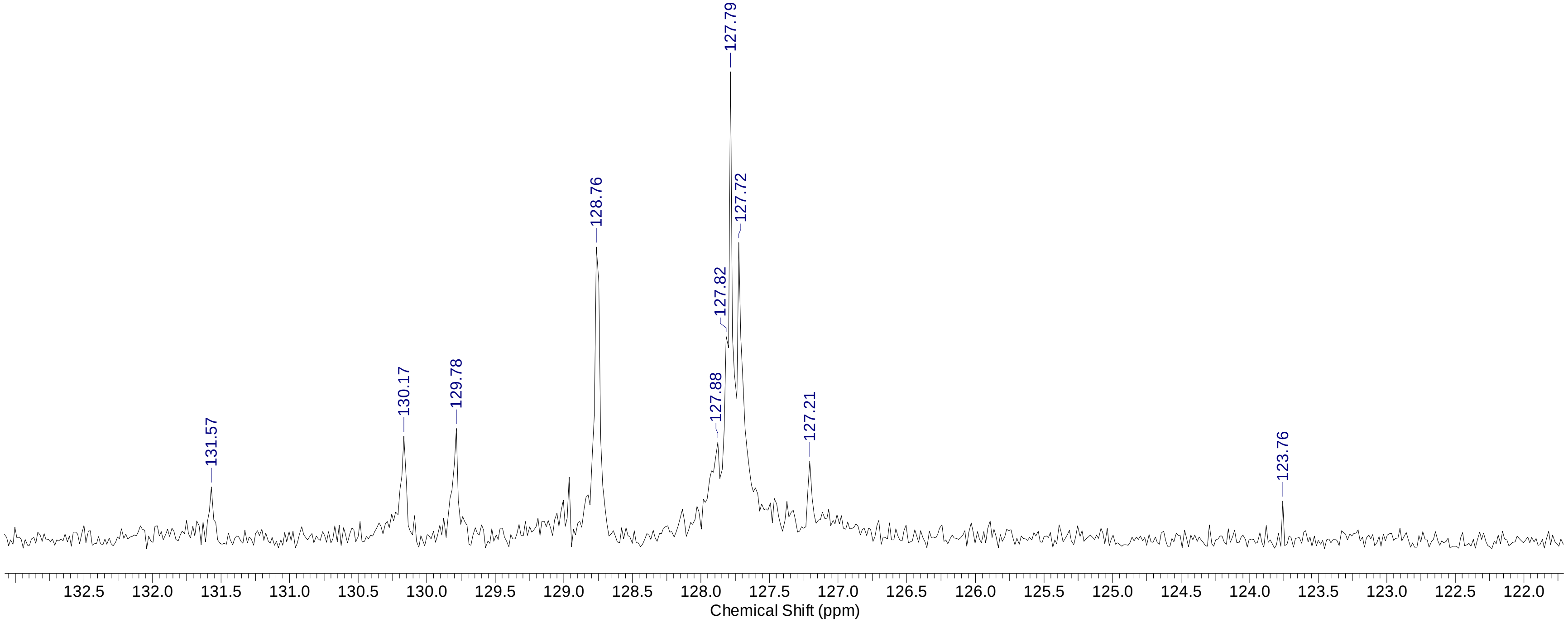

131.57
130.17
129.78
128.76
127.88
127.82
127.79
127.72
127.21
123.76
132.5
132.0
131.5
131.0
130.5
130.0
129.5
129.0
128.5
128.0
127.5
127.0
126.5
126.0
125.5
125.0
124.5
124.0
123.5
123.0
122.5
122.0
Chemical Shift (ppm)

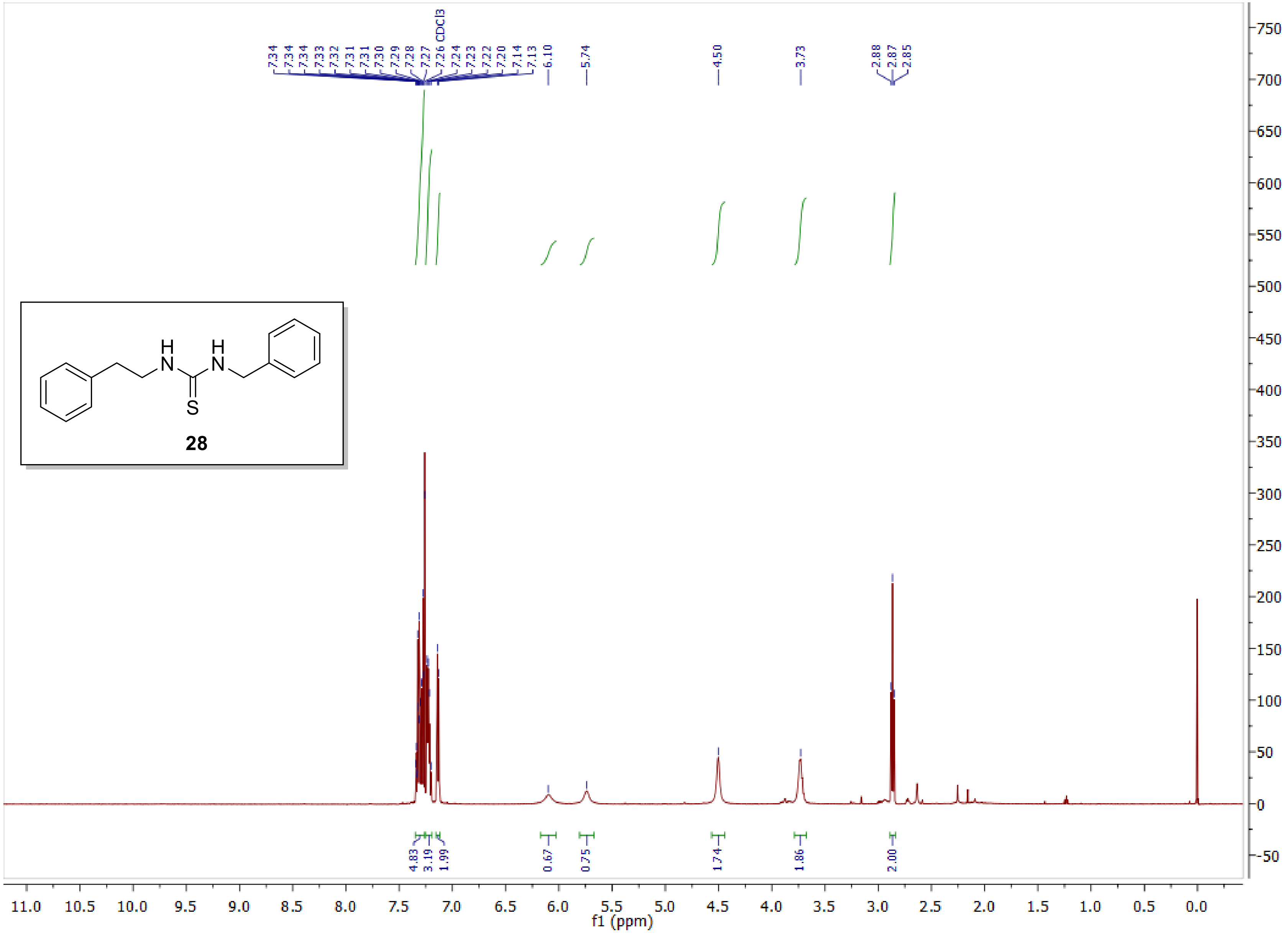
7.34
7.34
7.34
7.33
7.32
7.31
7.31
7.30
7.29
7.28
7.27
7.26 CDCl3
7.24
7.23
7.22
7.20
7.14
7.13
6.10
5.74
4.50
3.73
2.88
2.87
2.85
4.83
3.19
1.99
0.67
0.75
1.74
1.86
2.00
f1 (ppm)
H
N
H
N
S
28

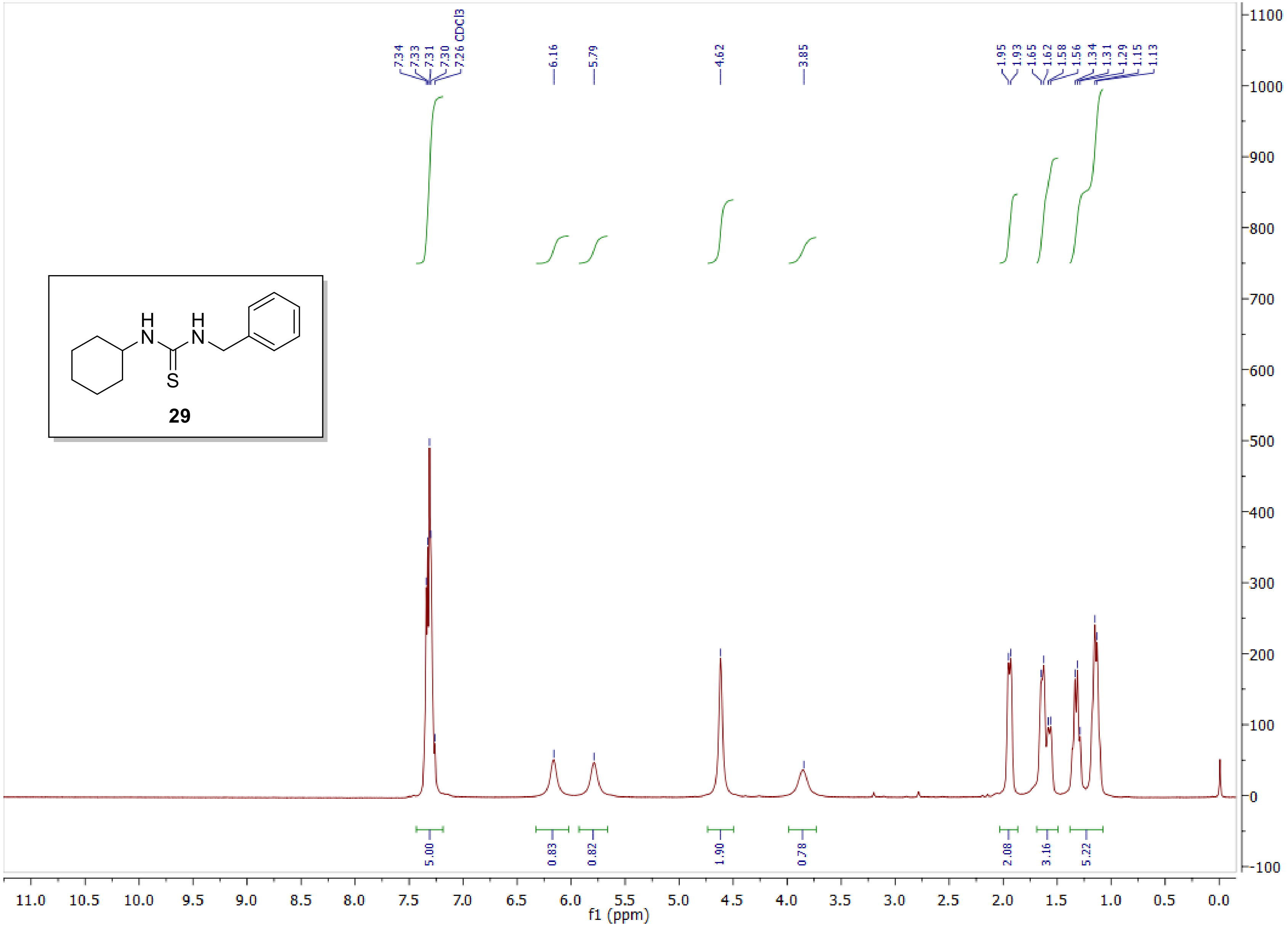
7.34
7.33
7.31
7.30
7.26 CDCl3
6.16
5.79
4.62
3.85
1.95
1.93
1.65
1.62
1.58
1.56
1.34
1.31
1.29
1.15
1.13
5.00
0.83
0.82
1.90
0.78
2.08
3.16
5.22
f1 (ppm)
29

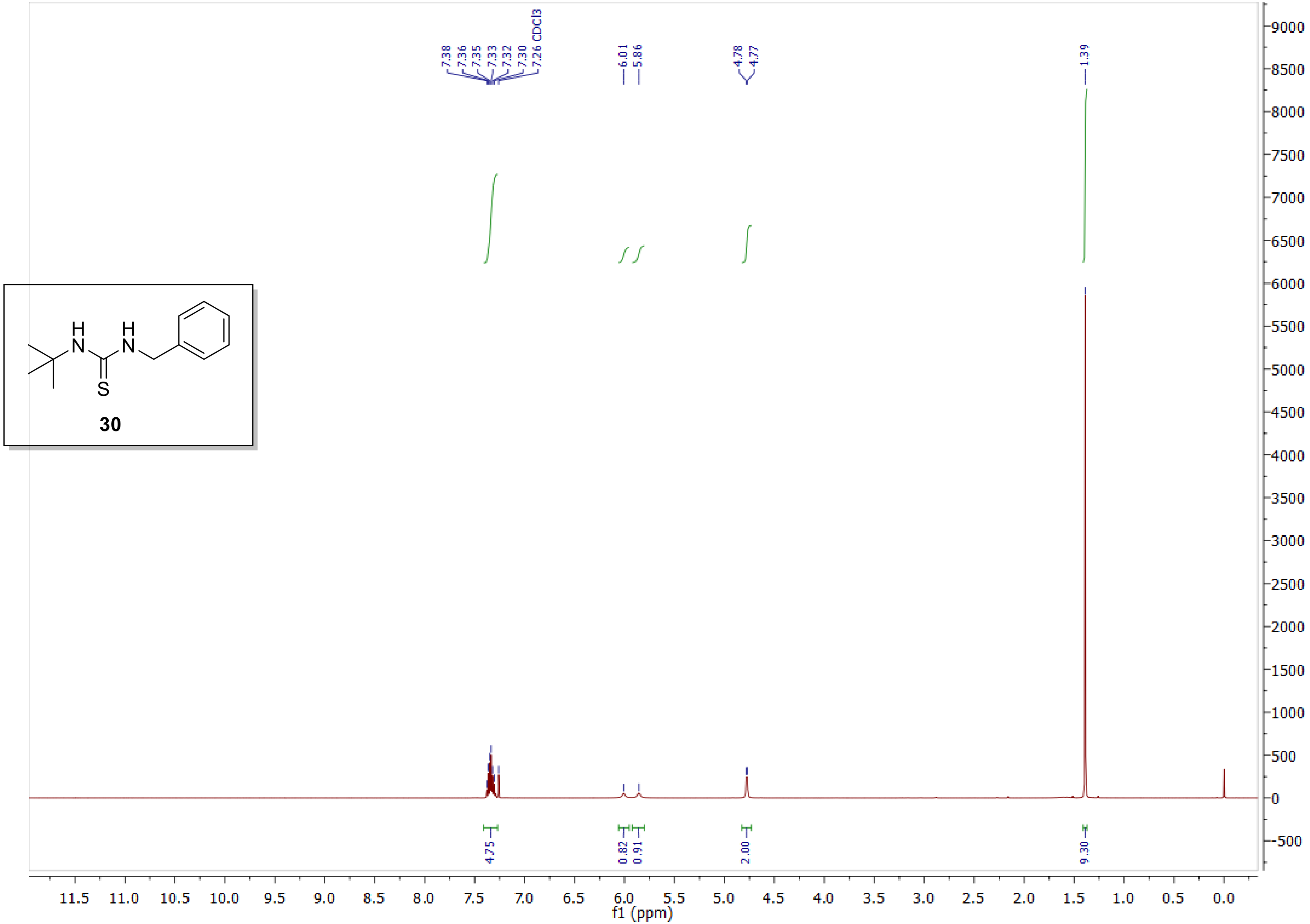
7.38
7.36
7.35
7.33
7.32
7.30
7.26 CDCl3
6.01
5.86
4.78
4.77
1.39
4.75
0.82
0.91
2.00
9.30
f1 (ppm)
H
N
H
N
S
30

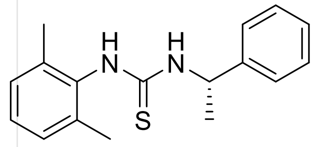

31

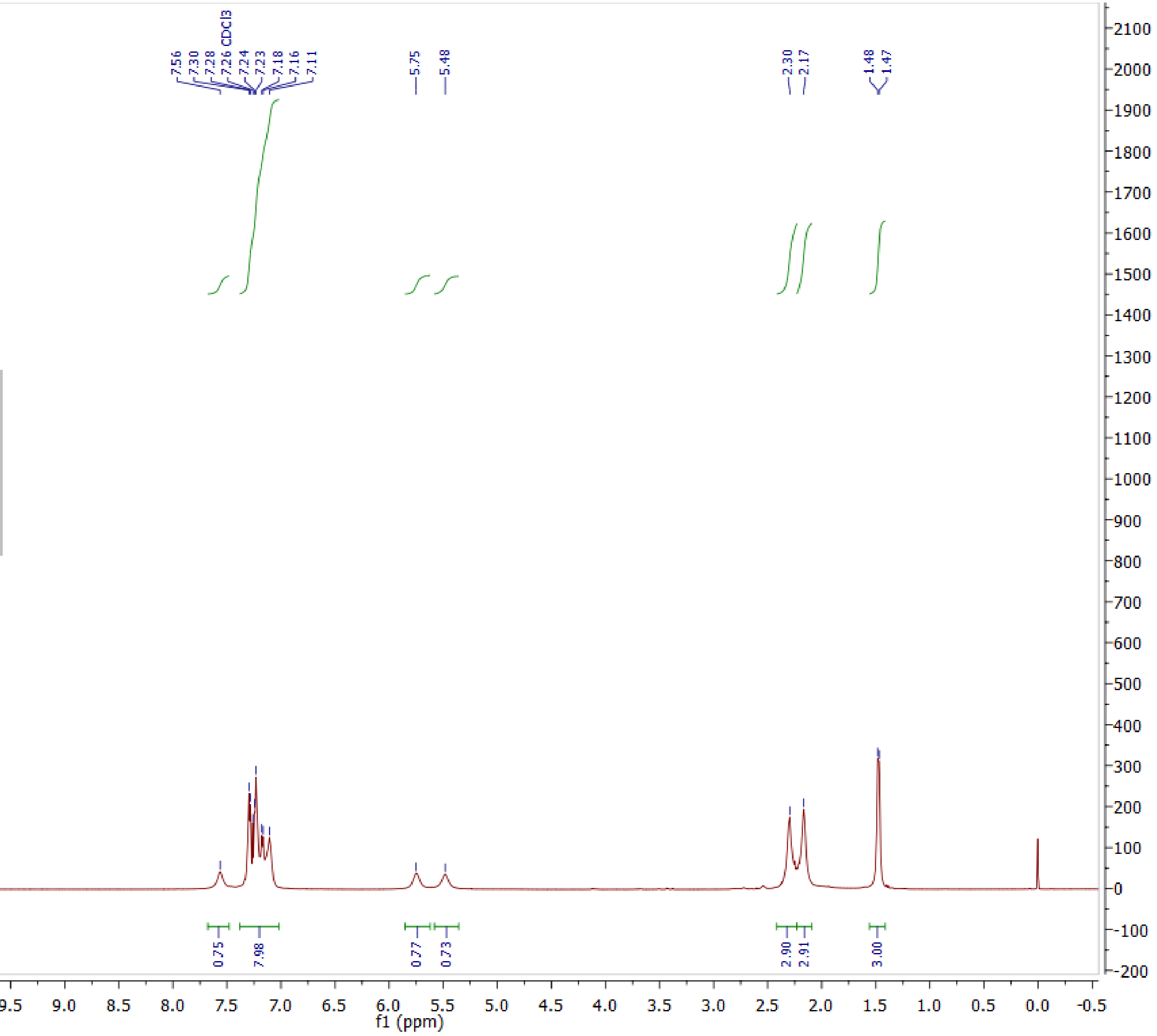

7.56
7.30
7.28
7.26 CDCl3
7.24
7.23
7.18
7.16
7.11
5.75
5.48
2.30
2.17
1.48
1.47
0.75
7.98
0.77
0.73
2.90
2.91
3.00
f1 (ppm)

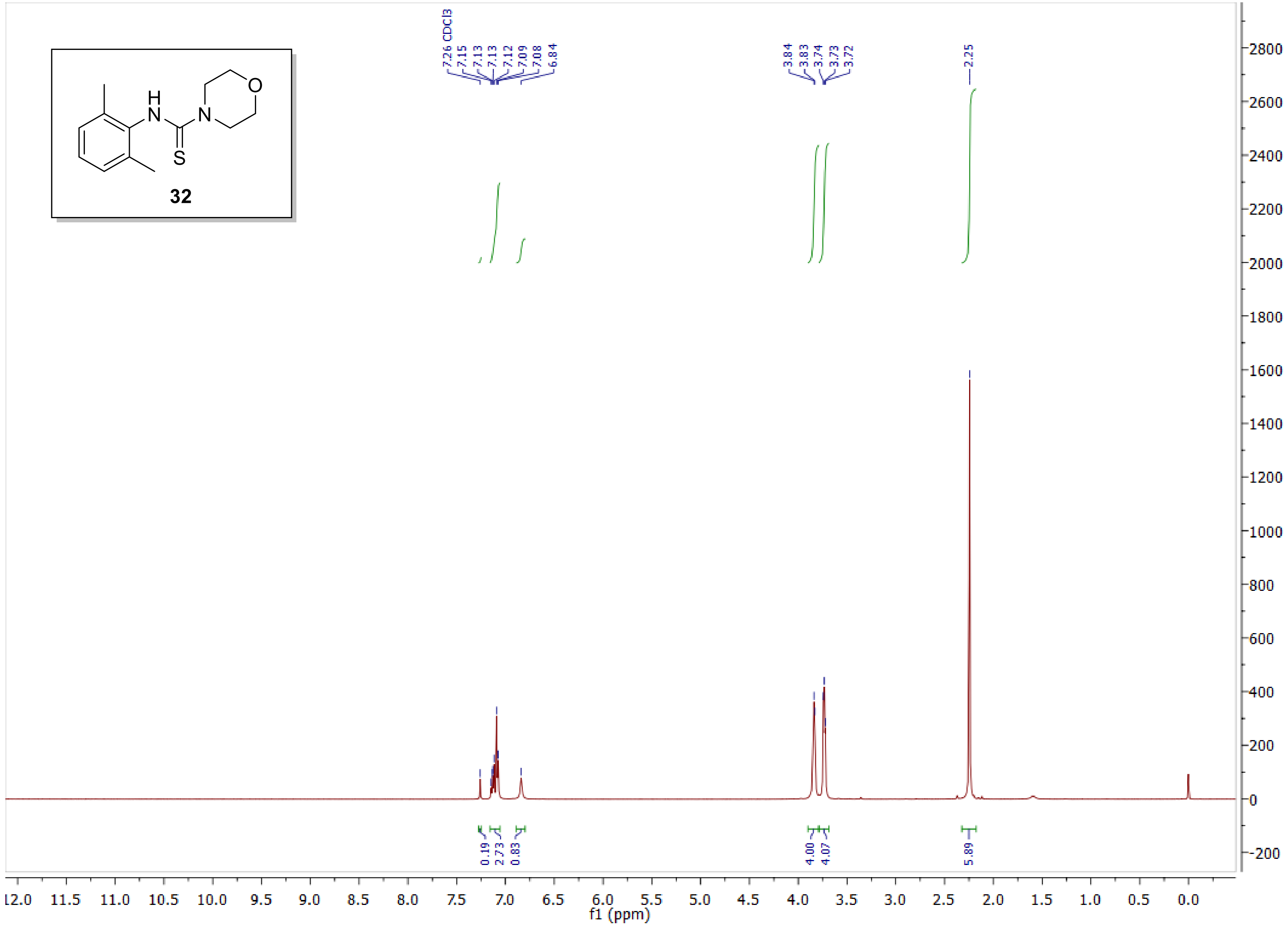

32
7.26 CDCl3
7.15
7.13
7.13
7.12
7.09
7.08
6.84
3.84
3.83
3.74
3.73
3.72
2.25
0.19
2.73
0.83
4.00
4.07
5.89
f1 (ppm)

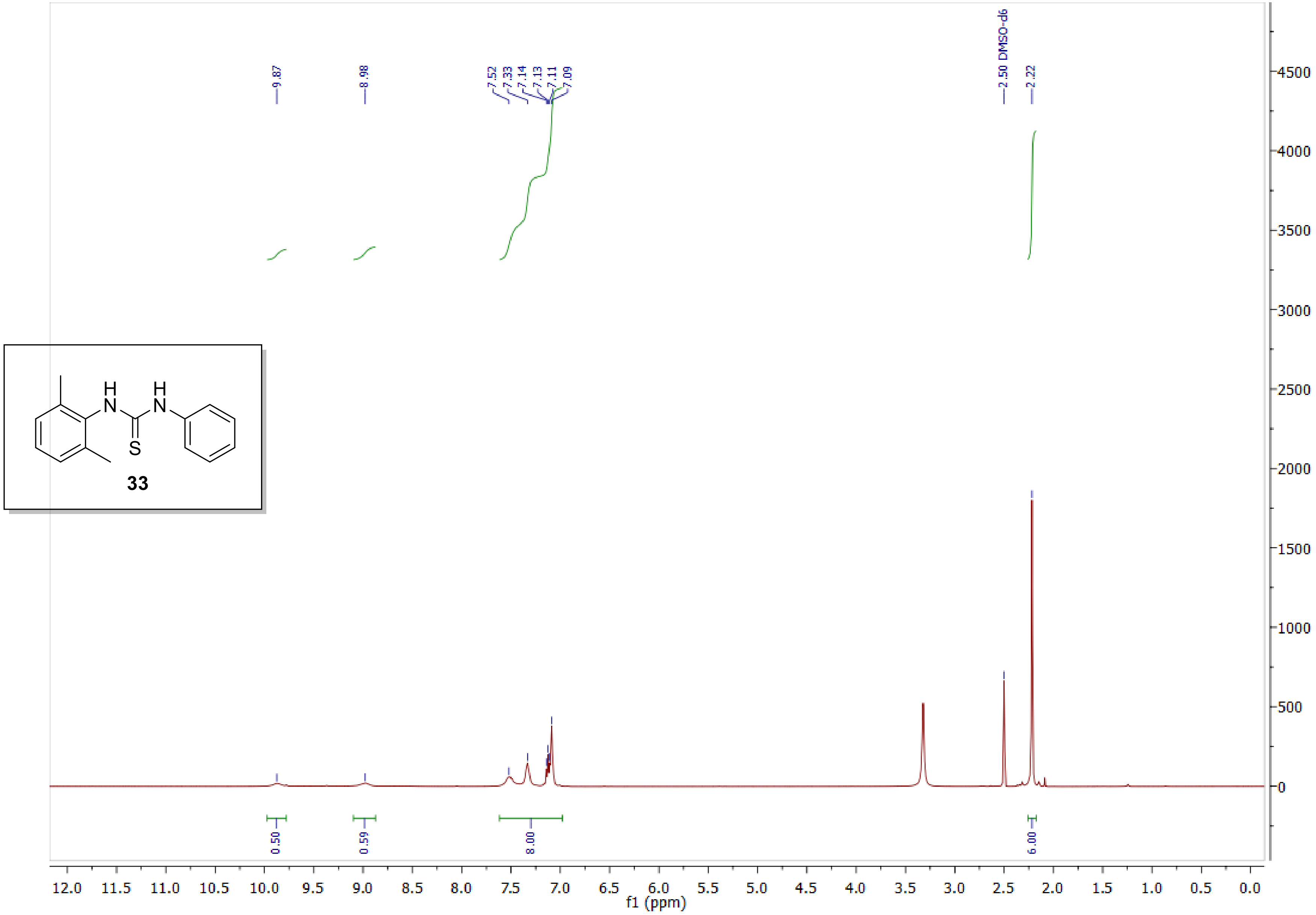
9.87
8.98
7.52
7.33
7.14
7.13
7.11
7.09
2.50 DMSO-d6
2.22
0.50
0.59
8.00
6.00
f1 (ppm)
33

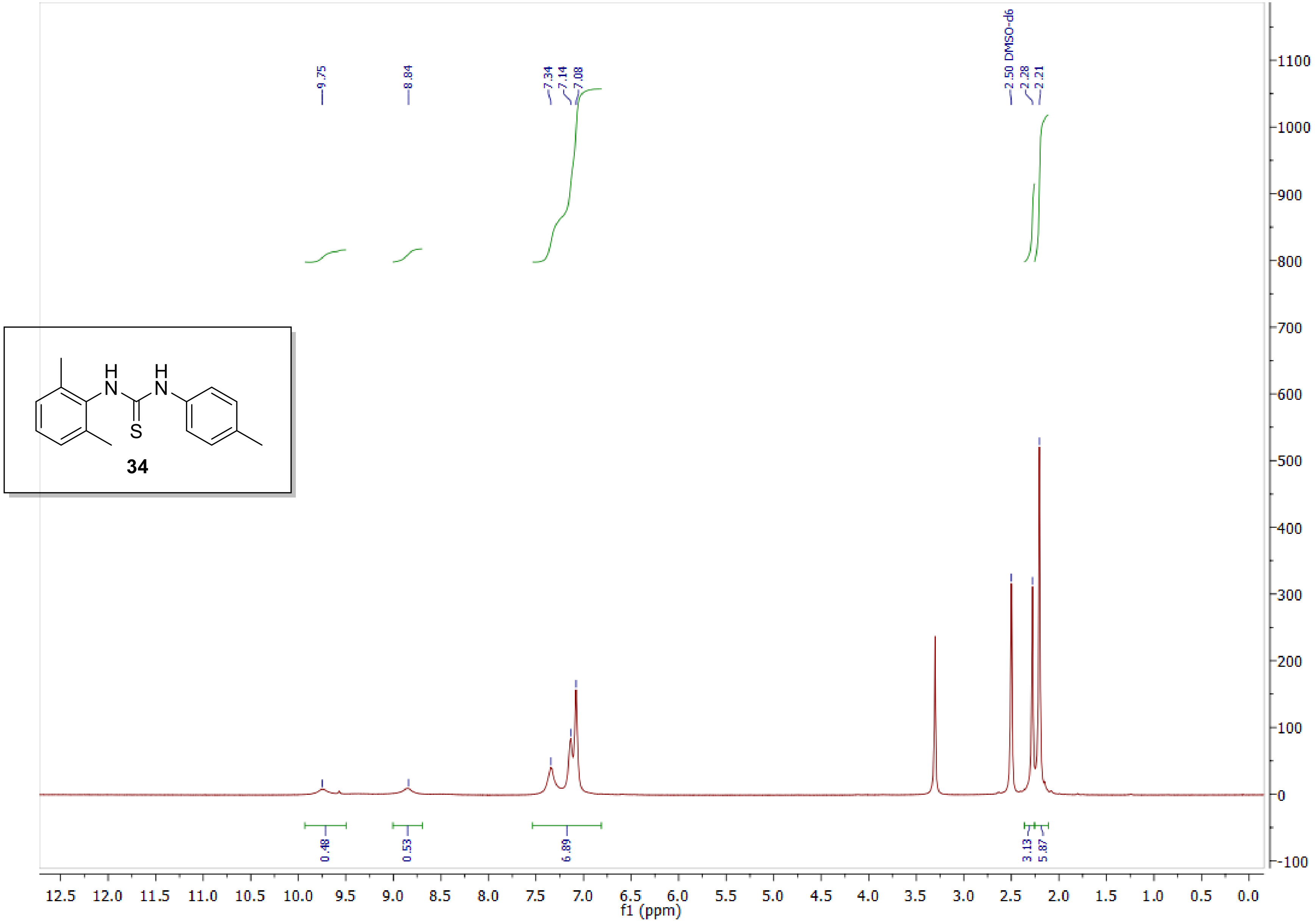

9.75
8.84
7.34
7.14
7.08
2.50 DMSO-d6
2.28
2.21
34
0.48
0.53
6.89
3.13
5.87
12.5 12.0 11.5 11.0 10.5 10.0 9.5 9.0 8.5 8.0 7.5 7.0 6.5 6.0 5.5 5.0 4.5 4.0 3.5 3.0 2.5 2.0 1.5 1.0 0.5 0.0
f1 (ppm)
1100 1000 900 800 700 600 500 400 300 200 100 0 -100

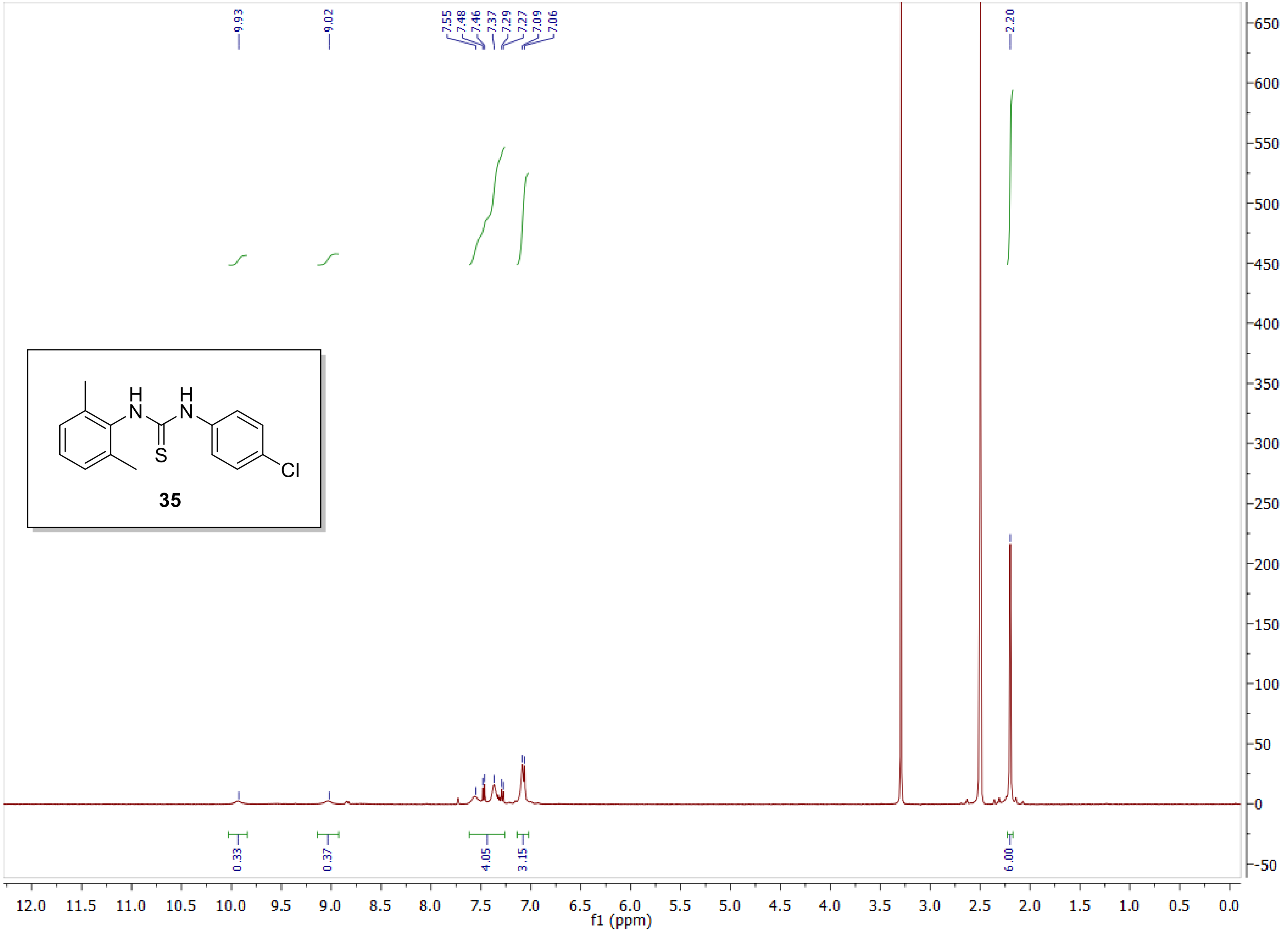
9.93
9.02
7.55
7.48
7.46
7.37
7.29
7.27
7.09
7.06
2.20
35
Cl
S
0.33
0.37
4.05
3.15
6.00
f1 (ppm)

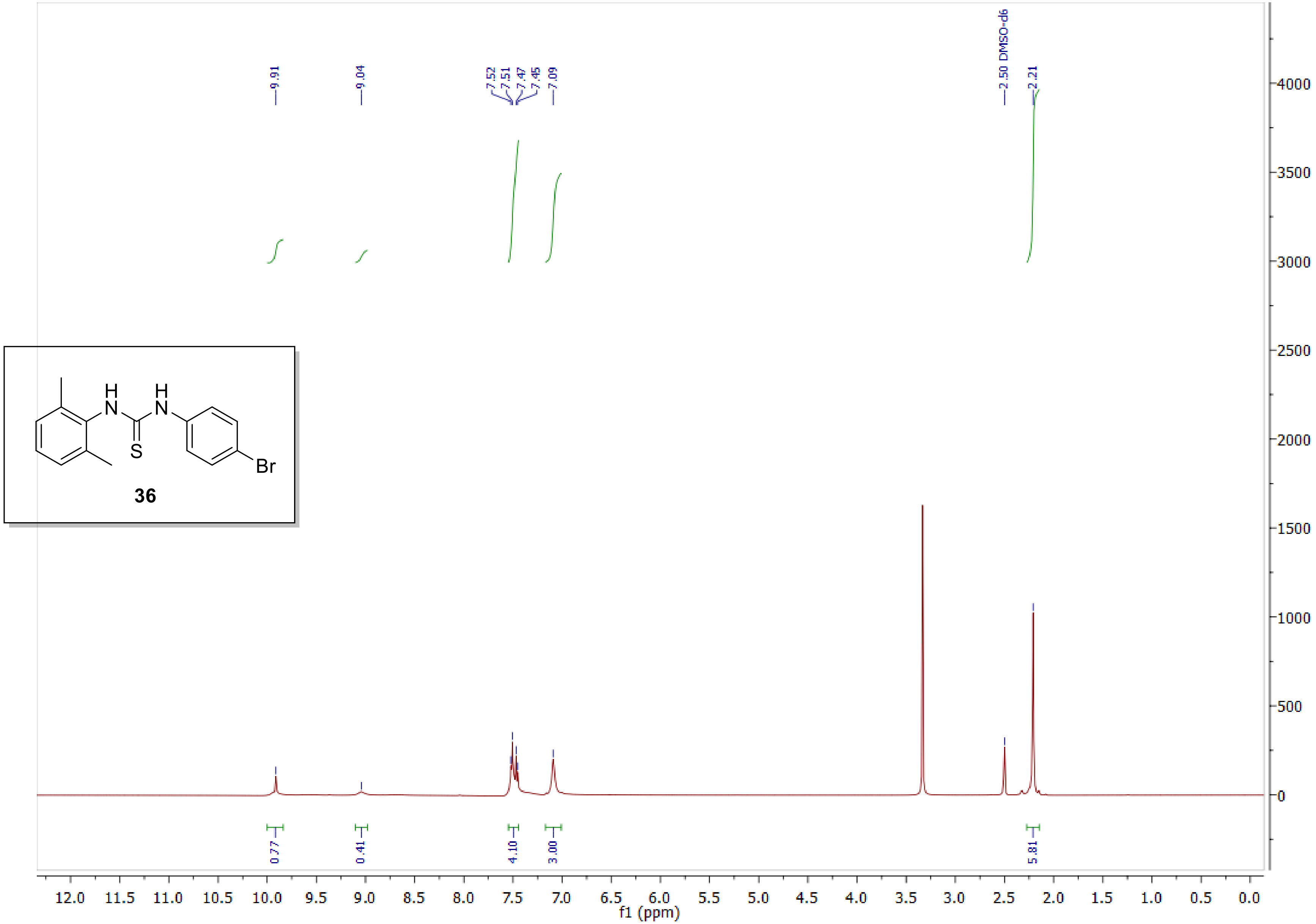
9.91
9.04
7.52
7.51
7.47
7.45
7.09
2.50 DMSO-d6
2.21
0.77
0.41
4.10
3.00
5.81
4000
3500
3000
2500
2000
1500
1000
500
0
12.0 11.5 11.0 10.5 10.0 9.5 9.0 8.5 8.0 7.5 7.0 6.5 6.0 5.5 5.0 4.5 4.0 3.5 3.0 2.5 2.0 1.5 1.0 0.5 0.0
f1 (ppm)
H
N
H
N
S
Br
36

References:

1. Németh, A. G., Szabó, R., Domján, A., Keserű, G. M. & Ábrányi-Balogh, P. *ChemistryOpen* (2020) https://doi.org/10.1002/open.202000250.
2. Ikawa, T., Barder, T. E., Biscoe, M. R. & Buchwald, S. L. *J. Am. Chem. Soc.* **129**, 13001–13007 (2007).
3. Chapman, R. S. L., Lawrence, R., Williams, J. M. J. & Bull, S. D. *Org. Lett.* **19**, 4908–4911 (2017).
4. Okandeji, B. O., Greenwald, D. M., Wroten, J. & Sello, J. K. *Bioorg. Med. Chem.* **19**, 7679–7689 (2011).
5. Grolla, A. A. *et al. J. Med. Chem.* **52**, 2776–2785 (2009).
6. Günay, N. S., Cihan, G., Kocabalkanli, A. & Çapan, G. *Marmara Pharm. J.* **15**, 100–104 (2011).
7. Nguyen, T. B., Ermolenko, L. & Al-Mourabit, A. *Synth.* **46**, 3172–3179 (2014).
8. Li, T. T., Song, X. H., Wang, M. S. & Ma, N. *RSC Adv.* **4**, 40054–40060 (2014).
9. Mohanta, P. K., Dhar, S., Samal, S. K., Ila, H. & Junjappa, H. *Tetrahedron* **56**, 629–637 (2000).
10. Custelcean, R., Gorbunova, M. G. & Bonnesen, P. V. *Chem. - A Eur. J.* **11**, 1459–1466 (2005).
11. Varun, B. V. & Prabhu, K. R. *RSC Adv.* **3**, 3079–3087 (2013).
12. Jen, T., Hoeven Van, H., Groves, W., McLean, R. A. & Loev, B. *J. Med. Chem.* **18**, 90–99 (1975).
13. Krasovskiy, Arkady, L. & Szuromi, E. US2019/263949A1 (2019).
